# Clinical translation of handheld optical coherence tomography: practical considerations and recent advancements

**DOI:** 10.1117/1.JBO.22.12.121715

**Published:** 2017-12-19

**Authors:** Guillermo L. Monroy, Jungeun Won, Darold R. Spillman, Roshan Dsouza, Stephen A. Boppart

**Affiliations:** a Beckman Institute for Advanced Science and Technology, Urbana, Illinois, United States; b University of Illinois at Urbana-Champaign, Department of Bioengineering, Urbana, Illinois, United States; c University of Illinois at Urbana-Champaign, Department of Electrical and Computer Engineering, Urbana, Illinois, United States; d Carle-Illinois College of Medicine, Urbana, Illinois, United States

**Keywords:** optical coherence tomography, handheld, diagnostics, point-of-care, system development, multimodal

## Abstract

Since the inception of optical coherence tomography (OCT), advancements in imaging system design and handheld probes have allowed for numerous advancements in disease diagnostics and characterization of the structural and optical properties of tissue. OCT system developers continue to reduce form factor and cost, while improving imaging performance (speed, resolution, etc.) and flexibility for applicability in a broad range of fields, and nearly every clinical specialty. An extensive array of components to construct customized systems has also become available, with a range of commercial entities that produce high-quality products, from single components to full systems, for clinical and research use. Many advancements in the development of these miniaturized and portable systems can be linked back to a specific challenge in academic research, or a clinical need in medicine or surgery. Handheld OCT systems are discussed and explored for various applications. Handheld systems are discussed in terms of their relative level of portability and form factor, with mention of the supporting technologies and surrounding ecosystem that bolstered their development. Additional insight from our efforts to implement systems in several clinical environments is provided. The trend toward well-designed, efficient, and compact handheld systems paves the way for more widespread adoption of OCT into point-of-care or point-of-procedure applications in both clinical and commercial settings.

## 1 Introduction

Advancements in the field of biophotonics, specifically in optical coherence tomography (OCT) imaging, have allowed for numerous discoveries in disease diagnostics and treatment applications in both commercial and clinical applications, driven forward by academic research and development. OCT first provided an optical means to generate noninvasive high-resolution cross-sectional images and depth-resolved measurements of the human eye.^1^^–^^3^ OCT system development today has continued to reduce form factor and cost, while improving on imaging performance (speed, resolution, etc.) and offering more flexibility for applications in a variety of clinical subspecialties beyond ophthalmology.

OCT has followed a similar development trajectory^4^ as that of other clinically useful medical imaging techniques, albeit at a much more accelerated pace. Ultrasound (US) imaging has an extensive history of development, with its inception sometime around the 1920s, and impactful clinical utility demonstrated in approximately 1958.^5^ US systems as understood today were first released commercially in cart-based systems around 1980, with improvements in form factor, utility, and capability over time to its present appearance as a compact, well designed, and optimized system for specialized clinical exams.^6^ Recently, ultracompact, battery powered, and completely self-contained US handheld units have been released that are used in routine checkups, low-resource settings, and emergency situations (EMT type).^7^ Over a span of ∼60 years, US became recognized as the gold-standard imaging modality for a wide range of clinically useful diagnostic tests.

In comparison, the rate of development and clinical adoption of OCT has exceeded that pace, given the acceptance of OCT into the ophthalmic community^8^ as a gold-standard technique for retinal evaluation ∼20 to 25 years after inception. The adoption of OCT into the clinical pathways of ophthalmology has allowed investigation into numerous pathologies,^9^^,^^10^ and the structure and function of the individual components of the eye, including the cornea,^11^^,^^12^ lens,^13^^,^^14^ iris,^15^^,^^16^ ciliary body,^17^^,^^18^ retina,^19^^–^^22^ microvasculature,^23^^,^^24^ contact lens design and fit,^25^^,^^26^ and even changes in the retina with exposure to space flight.^27^ It is often more effective to use a desktop- or benchtop-based system for these high-resolution commercial systems, since they usually require more precise optical stability during imaging to provide high-quality images,^28^ or have complex feedback loops, such as in adaptive optics systems.^29^^,^^30^

Still, there are many use-cases where benchtop systems have been adapted to provide more advantageous and flexible imaging in probe-like units for subjects who do not have sufficient mobility to tolerate a typical eye exam. Examples of these situations range from bedridden patients to life science and animal research subjects, especially when the temperament of the animal or simply its physical size prohibits bringing it to the imaging equipment. The needs of end users have inspired engineers to package full systems into compact, portable carts. These efforts have advanced with the supporting OCT ecosystem^31^ of vendors, original equipment manufacturers (OEMs), and machine shops.

In this review, the development of handheld OCT systems for applications including and beyond ophthalmology, as well as the supporting technologies and techniques that have helped engineers produce these systems, will be discussed. We also share a more practical overview and perspective of the handheld probe and portable system development from our own group, with specific lessons learned and pitfalls to avoid when developing and using systems use in clinical settings. Next, the overall field of research and development of handheld OCT probes is presented along with a review of the literature, organized by the relative level of system portability, and with specific systems highlighted from most all imaging subspecialties. Finally, next-generation technologies are discussed that will further reduce handheld probe form factor and allow for future handheld integrated systems.

## 2 Innovative Engineering and the OCT Ecosystem

The capabilities of the numerous OCT systems that exist today^32^ allow for broad applicability, ranging from commercial and clinical applications to basic science research. These advancements were able to iterate so quickly, in part, due to the wide commercial availability of hardware, and the development of miniaturized optical components.^33^ The incredible rate of growth and expansion of OCT^34^ has greatly benefited from the development trends in other fields, such as the optical telecommunications field, providing improvements in fiber optics, detectors, and light sources. Present day, expertise in design and manufacturing of miniaturized and specialized components, along with the rapid availability of nearly any optical component, allows for various approaches to construct an optical system to fit a specific need. Clinical-quality components from OEM and third party vendors allow researchers to integrate components such as endoscopes and catheters directly and provide a means to produce high-quality and clinically focused research systems.

While the semiconductor industry lends high-performance detection and processing technology that has reduced cost and improved capability, software development also provides improvements in image detection,^35^^–^^37^ image quality, and signal-to-noise ratio (SNR),^38^^–^^40^ through techniques such as digital refocusing in interferometric synthetic aperture microscopy,^41^^,^^42^ computational (computer-based) adaptive optics,^43^ or functional extension techniques such as Doppler and elastography.^44^ The aforementioned availability of components and hardware, paired with the advances in digital optical design, project management, precision capability at machine shops, and three-dimensional (3-D) printing and computer-aided design (CAD) programs, enables engineers to design and implement a high-quality system.

**Fig. 1:**
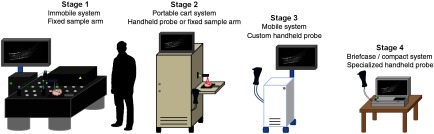
Visual representation of stages 1 to 4 OCT systems. As system form factor is reduced, tradeoffs between image quality (SNR, phase stability, resolution, weight, and scan speed) and portability must be managed. For later-stage systems, reducing cost becomes more of a concern as well. Eventually, image quality and system capabilities become more focused on meeting the needs of a clinical application or a commercial product at a specific price point.

To aid in later discussion throughout this review, OCT systems can be stratified into different stages, as demonstrated in Fig. 1, by the level of system portability and form factor.

Stage 1: Immobile OCT system + fixed sample arm
  These systems occupy significant real estate on an optical (floating) table, typically using a stationary scan stage or mount to image samples. Processing is performed on a large desktop computer and monitor. (Not the focus of this review.)
Stage 2: Portable OCT system + tethered handheld probe
  The OCT system, image processing unit, and display are self-contained within a portable cart. An optical breadboard holds and stabilizes optical components in the cart and can be easily reconfigured. The system power supply is not self-contained and requires an external power source (wall outlet). The handheld probe is likely built using mostly off-the-shelf components, but can be packaged into commercial-grade enclosures.
Stage 3: Mobile OCT system + handheld probe
  The entire OCT system and tethered handheld probe are completely self-contained (acquisition, processing, display, and rechargeable power source) in a specialized portable cart or system and customized for a single application. Optimizations to reduce weight and form factor may include customized (field-programmable gate array or similar) processing units and imaging hardware. Tradeoffs with scan speed, resolution, or image quality may be more noticeable than in earlier stages at the expense of form factor and cost.
Stage 4: Briefcase-sized (or smaller) self-contained handheld OCT system
  A compact, handheld, battery-powered system contains all optical components, processing units, and a display in a form factor no larger or heavier than a small briefcase. The handheld probe hardware and operating software are completely customized for a single application, with basic online processing to ensure reduced power consumption. Robust system packaging meets electrical, liquid, mechanical, and chemical safety specifications for full clinical certification and rigorous daily use.

Building handheld systems is truly an iterative process, not only for the hardware and software to operate the system but also to define the essential capabilities of a system and how the handheld system is to be used in practice. In recent years, the performance of equipment relative to size and footprint has rapidly improved, reducing the impact of tradeoffs encountered with designing systems. The discussion then often turns to cost and flexibility of hardware and software. However, the motivation to develop a portable system often arises from needs in the research applications work flow. In the next section, we provide insight into this process, how challenges and needs in research projects have shaped the needs for portable systems, and in turn, how these were shaped by the OCT community and ecosystem.

## 3 An Iterative Process: Insight in Designing, Building, and Translating Handheld OCT Systems for Clinical Use

A major focus of translational research has been in bringing optical imaging systems and devices out of the lab and into a hospital or clinical setting. Over time, with changing needs from new research projects and specific challenges that arise from the clinical environment, as well as with hardware improvements and availability of components, systems have advanced from lab-based Stage 1 systems to portable Stage 2 systems. Here, some specific clinical applications and practical insight is provided based on experiences in the development and progress toward Stage 2 portable OCT systems.

### 3.1 Intraoperative Assessment of Cancerous Tissue

Breast cancer, the most common cancer women face worldwide (apart from skin cancer) has a projected cost increase for diagnosis and care by at least 30% over the next 3 years.^45^ However, costs can be significantly reduced through early detection and diagnosis of breast cancer.^46^ There are numerous procedures available to detect breast cancer, including the physical palpation of breast tissue or noninvasive medical imaging of the breast using mammography (x-ray), US, or magnetic resonance imaging (MRI). After detection, diagnosis is confirmed through the histological analysis of suspicious tissue following biopsy. With a confirmed diagnosis, the extent (stage) of the cancer and the corresponding appropriate treatment options are determined.^47^ The surgical removal of the tumor is a mainstay treatment option of the disease.^48^^,^^49^

**Fig. 2:**
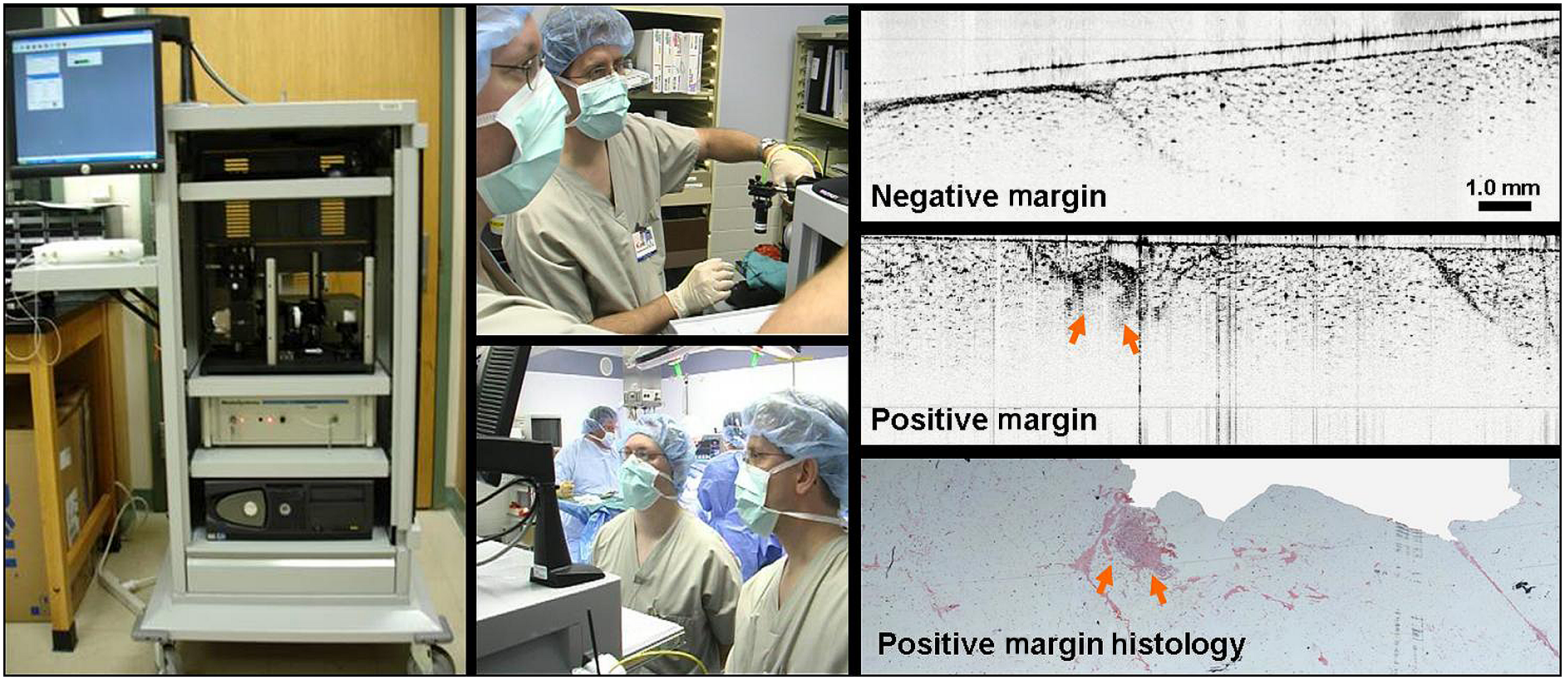
First-generation intraoperative portable spectral-domain OCT clinical system—“The Beast.” OCT images of *ex vivo* resected tumor masses provide rapid and label-free discrimination of surgical tumor margins. Intraoperative OCT images were correlated and verified with postoperative histology.^51^

A major problem faced by surgical oncologists when removing cancerous tissue is ensuring that all cancerous tissue is removed, as well as a sufficient buffer, or margin, of potentially reactive tissue around the tumor mass. There is a high rate of re-excision surgeries for breast cancer given the difficulty of appropriately removing the right amount of tissue, especially in breast-conserving surgeries.^50^ One of the first portable intraoperative OCT systems was contained in a cart aptly named “The Beast” due to its size. The long-term goal for this research and system was to give real-time intraoperative feedback to surgeons by assessing the surgical margin of resected breast lumpectomy specimens. Figure 2 depicts the system, enclosed in a standard medical-grade endoscopy cart, ∼5  ft tall, with a monitor affixed (hanging) off the top, and a small ($<2\;{\mathrm{ft}}^{2}$) drop shelf that served as the imaging platform. There was a single drawer where tools were kept, along with marking ink that was used to delineate the location of the imaging site for later histological correlations. This OCT system and cart were well received in the operating room, where images were seen in real-time by research, surgical, and nursing staff, many seeing OCT images of surgical margins for the first time. While state-of-the-art for its time, the spectral-domain OCT system was rather rudimentary by today’s standards. To produce cross-sectional and volumetric images, it utilized a scanning stage to translate fresh *ex vivo* tissue specimens. The system required up to 3 min for image acquisition, depending on the imaged tissue volume, with an axial scan rate of 5 kHz, and axial and transverse resolutions of ∼8 and 35  μm, respectively. This system, however, provided some of the first intraoperative OCT images from freshly resected tumor masses.^51^ The system and representative images of negative and positive margins are shown in Fig. 2.

### 3.2 Assessing Lymph Nodes for Metastatic Disease: System Design and Implementation

While evaluating surgical margins, another major investigational efforts focused on intraoperatively assessing lymph nodes (LN) for the presence of metastatic disease, including micrometastases.^52^^,^^53^ LN resection and postoperative histological analysis is the current standard-of-care^54^ for clinically staging breast cancer. In practice, surgeons use an injectable dye to visually localize sentinel or axillary LNs, sometimes using preoperative imaging (US, positron-emission tomography) as a guide. Similarly, radioactively labeled tracers, along with a gamma/Geiger counter probe, can be used for intraoperative localization. Locating and removing the sentinel LN(s) allow pathologists to determine whether there has been metastatic progression of cancer. However, this process is notoriously time-consuming and involves postoperative histopathology of thin sections throughout the entire resected LN. The removal of LNs must also be carefully balanced against the risk of lymphedema^55^ and other long-term complications. Along with other biophotonics researchers, we realized that an intraoperative noninvasive analysis of LNs could provide real-time guidance to surgeons, and in the future, potentially reduce or even prevent unnecessary excision of LNs.

**Fig. 3:**
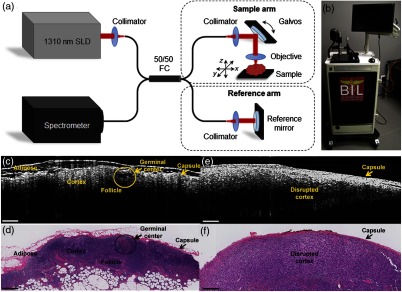
Second-generation intraoperative portable spectral-domain OCT clinical system. (a) OCT system schematic and (b) photo of the OCT system. Physical dimensions were greatly reduced over the previous version. (c, e) Representative intraoperative OCT and (d, f) corresponding histopathology images of a normal, nonmetastatic (left) and cancerous metastatic (right) *ex vivo* human LNs. All scale bars represent 0.5 mm. Modified and reprinted with permission.^53^

While preliminary investigations into LNs were carried out with “the Beast,” a second-generation cart, shown in Fig. 3, was developed and used for the intraoperative assessment of LNs. This system employed one of the first commercial benchtop OCT systems (Bioptigen) that included a handheld probe. This system and probe contained both a pair of integrated scanning galvanometers for 3-D image acquisition and a visible tracking beam and was retrofitted into a portable cart. Although the system had a handheld probe, it was used primarily in a fixed platform configuration with the probe mounted on a three-axis stage to both translate the probe over large distances across large tissue specimens and reduce motion artifacts. With this system, the assessment of surgical margins and LNs continued and expanded the previous study in scope, sample types, and analysis methods. When compared to the previous system, the improved imaging speed and overall usability of this system significantly increased sample interrogation capabilities within the operating theater. The integrated tracking beam was a key feature that helped ensure that the cross-sectional OCT images could be later correlated with the corresponding histological sections.^53^ This system also allowed for a simpler preparation protocol before entering into the surgical suite, as there was simply less surface area to wipe down and sterilize. Many of the materials used in this system were also protected against rust, which prevented its appearance and operation from significantly degrading over time.

### 3.3 Considerations for In Vivo Imaging: Protocols and System Design

Through these initial intraoperative studies, it was realized that this imaging technology could have its greatest impact by enabling a surgeon to determine if any cancerous tissue was left behind in the *in vivo* tumor resection bed (tumor cavity) following the removal of the tumor mass. However, achieving this goal would require a completely new system, handheld probe, and imaging protocol to image *in situ* in a human subject. New procedures were developed and adopted to avoid causing delays due to added research activities in the operating room, including system setup, usage, and imaging protocols that may otherwise unnecessarily increase the time the patient was anesthetized.

**Fig. 4:**
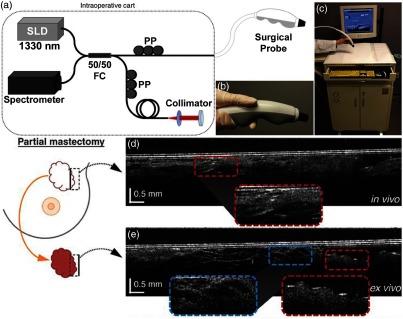
Third-generation intraoperative portable OCT system and handheld probe. (a) System schematic and (b) handheld probe, when used with surgical sheath, can be used intraoperatively and *in situ*. (c) Handheld probe and OCT system in portable cart. (d) *In vivo* images from a partial mastectomy margin. (e) *Ex vivo* confirmation of conjugate side. OCT images were correlated and confirmed with corresponding histology. Reprinted with permission.^56^

Following typical surgical protocols, the OCT imaging procedure involved first passing the handheld probe to the surgical nursing staff to drape the probe in a sterile sheath, and then to the surgeon and into the sterile field to scan the resection bed margin *in situ*. After scanning, and while the surgeon was working toward completing the surgery and closing the incision, the probe was passed back to the research staff to scan the excised tissue sample outside of the sterile surgical field. If cancer was suspected during either imaging scenario, it was possible to conclude that insufficient tissue was removed. However, because the OCT system, probe, and protocol were not approved for clinical decision making, the collaborating surgeons were not able to act on any intraoperative imaging results. In the near future, it is expected that it will be possible to identify and immediately remove any additional cancerous tissue from the *in vivo* tumor cavity and resection bed. This project marked one of the first *in vivo* and *in situ* intraoperative use of a handheld OCT device inside human subjects.^56^ The system, probe, and representative data are shown in Fig. 4. This study required many hours of not only technology development and research but also in-depth collaboration with hospital staff to finalize and approve the imaging protocol, and establish the logistics and flow of new procedures in the operating room. For *in vivo* imaging studies such as these, it is essential to begin a discussion with clinical partners as early as possible, to not only gauge interest but specifically to learn operational norms and restrictions that are present in clinical environments.

The handheld probe shown in Fig. 4 was acquired through an NIH-supported collaboration with a commercial partner who had acquired FDA approval for the use of their own system. The probe was designed to be completely sealed, tested for electrical discharge, and certified to ensure safe use in human subjects. The probe design and rigorous safety testing greatly accelerated Institutional Review Board (IRB) approval, which otherwise presented a seemingly impassable regulatory hurdle. The cart selected for this intraoperative imaging system was based on those used by automotive technicians, providing storage space, a simple and inoffensive design, and a design suitable for robust daily use in challenging environments. As a required measure of safety, an isolation transformer was added to this system, and all future systems, to prevent any electrical discharge back into the hospital power system, or into our handheld probe or system, to significantly reduce risk to the operator or imaging subject. The transparent, sterile plastic sheath that allowed this probe to be used within the sterile surgical field and placed into a subject’s tumor cavity was vetted against multiple suppliers, as different plastic types often significantly and unpredictably reduced imaging quality. All of these provisions ensured that the standard of care would not be compromised and that the handheld OCT probe met the same sterility standards as other surgical instruments and clinical equipment after being inspected by the hospital Biomedical Engineering Department.

Building a portable imaging system with a handheld probe and fitting it into a small cart is a challenging task. Typically, this requires many iterations of system-wide re-examination to ensure that all components are compatible and design goals are met. One major design parameter was to ensure sufficient airflow in and throughout the cart, given the high-performance computer and optical detection electronics. In an effort to create a cart that could be easily sanitized by the typical wipe-down procedure required before entering into the surgical wing, several vents were initially eliminated on the cart. However, during use and without proper airflow, electronic equipment within the cart would easily overheat and become damaged after a short period of time, first encountered with the video card in the computer. The system was modified to include fan vents to increase airflow and remedy the issue, which was only possible because the sheet metal on the cart was able to accommodate the small loss in structural rigidity.

With this system, it was possible to intraoperatively evaluate surgical breast margins both *in situ* as well as from resected tissue specimens.^56^ The previous generation intraoperative systems were transferred for use in studies in veterinary surgical oncology.^57^ Ongoing intraoperative human imaging studies have expanded to include the construction and use of portable systems for polarization-sensitive OCT for enhanced tumor margin detection^58^^,^^59^ and a portable multiphoton multimodality imaging system for assessing the tumor microenvironment^60^ in the operating room. Although neither of these next-generation portable systems currently utilizes a handheld probe, this is an active area of technological development.

### 3.4 Optical Imaging in Point-of-Care Settings

In addition to the operating room, other clinical settings exist where real-time point-of-care imaging would be impactful. Primary care is one clinical specialty that is commonly overlooked by device manufacturers, primarily because the operating margins are very slim, and subsequently, only essential equipment can be purchased and utilized.^61^ However, primary care physicians are on the front line of care, seeing patients before referral to a specialist, and must be knowledgeable about a wide variety of injuries, diseases, infections, and community or societal trends. Physicians often rely on the physical exam and tools that typically only provide a subjective analysis of patient status. While there are significant advancements in laboratory diagnostic tests of blood, fluids, tissues, and in medical imaging, similar advancements have not commensurately followed for primary care. Physicians could more accurately detect and diagnose disease through the use of new tools to provide quantification of many of the conditions they regularly see and qualitatively assess, and more appropriately refer patients to specialists using quantitative evidence-based data.

In the primary care office, precedents of optical imaging instruments exist, including the ophthalmoscope to visualize the retina, and the otoscope to visualize the tympanic membrane (TM, or ear drum). The otoscope, a relatively simple magnifier and illuminator, is routinely used to diagnose otitis media (OM), a common infection of the ear in children. OM causes inflammation of the TM, and the development of an effusion, or fluid buildup within the middle ear, that causes pain and conductive hearing loss.^62^ Clinicians use the otoscope to visually interrogate the TM and middle ear to qualitatively identify subtle changes to discern infection. Due to the difficulty of accurately assessing the TM, it is challenging for even expert physicians, resulting in a diagnostic accuracy of ∼70%.^63^ While other techniques exist to diagnose OM, including tympanometry^64^ and pneumatic otoscopy,^65^ they are not regularly used or are difficult to perform correctly.^66^ Physicians could greatly benefit from a tool that provides specific and quantitative information related to OM. To treat OM, systemic antibiotics may be prescribed, although clinical standards regarding this are in flux.^67^ Still, OM is one of the most common reasons for a child to visit the primary care office worldwide,^68^ be prescribed antibiotics,^69^ and receive surgery under anesthesia.^62^ Yet, incorrect diagnoses can lead to the inappropriate prescription of antibiotics. This can subsequently lead to increased antibiotic resistance in a large portion of the population, which is a growing concern.^70^ To address this issue, new low-coherence interferometry (LCI)- and OCT-based handheld probes were developed to better detect, characterize, and quantify OM.

**Fig. 5:**
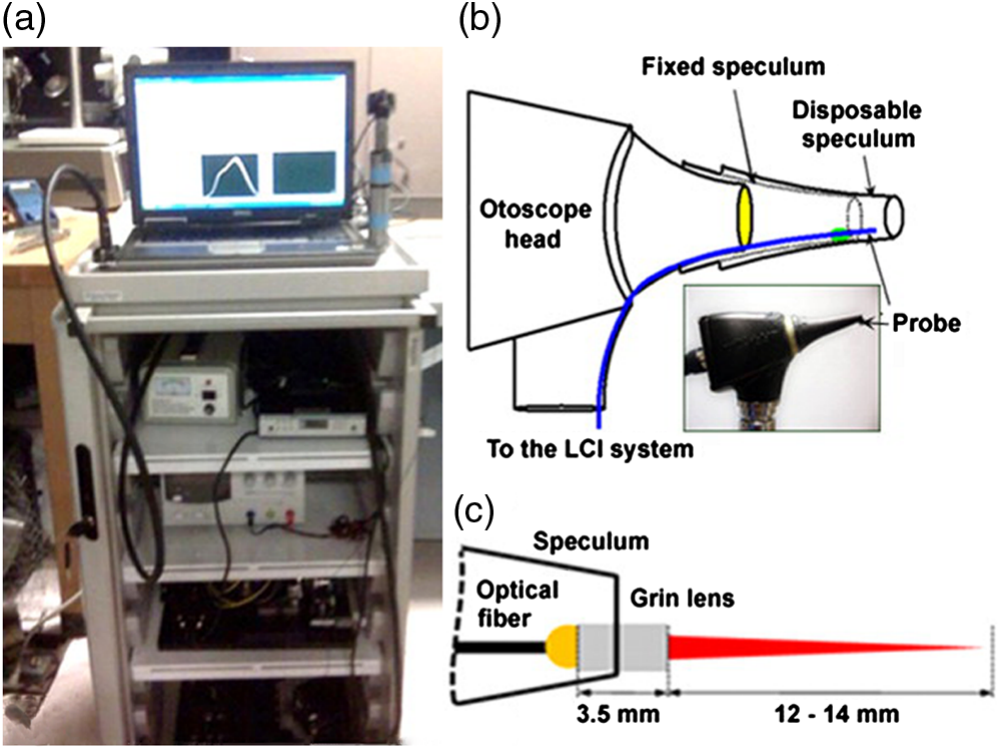
(a) Portable LCI-otoscopy system. (b) Schematic and photograph of the integration of the LCI fiber-based micro-optic probe into an ear speculum tip that would attach to the otoscope head. (c) Schematic and beam profile of the long-working-distance LCI probe, which used a gradient-index (GRIN) lens. Modified and reprinted with permission.^71^

In an initial design, a fiber and fused GRIN lens was added to the inside of a plastic ear speculum tip that attached to a commercial RCA-based video otoscope without modifying any of the internal components. Modifying an existing commercial product to expand functionality also greatly hastened the development of this system. The lens properties were chosen to focus light through the ear canal and onto and through the TM of a human subject. An LCI system that utilized a laptop for control and processing was constructed and placed into a portable cart for use in the otolaryngologist specialist office (Fig. 5). This system provided single A-scans at a rate of 35 kHz through the TM and into any content within the middle ear (effusion and biofilm). This system provided one of the first *in vivo* optical characterizations of the TM in an animal model^72^ and in adult human subjects,^71^ and helped develop a normative library of the depth-resolved scattering characteristics or profiles of various infection states for OM.

### 3.5 First-Generation Integrated Handheld Probe for Primary Care

Considering the regular use of both the otoscope and ophthalmoscope in the primary care office, it was realized that it would be possible to expand imaging to include the eye (both anterior and posterior), as well as the skin and oral cavity. A system with this expanded capability could improve upon not only the ophthalmoscope or otoscope but also provide a more comprehensive point-of-care imaging system for all the tissue sites commonly examined in primary care. While the ophthalmoscope can be used for this purpose, it only provides a surface view of the retina, and is difficult to use effectively. With the depth-resolved capabilities of OCT, an integrated system could provide more comprehensive ocular exams, or early screening for diabetic retinopathy (DBR), which slowly causes patients to lose their vision.^73^ Vision loss occurs through either the leakage of fluid into the macula, which eventually causes macular edema (nonproliferative DBR), or from the growth of new vessels on the retina with resultant scar tissue that restricts vision, and may eventually lead to a detached retina (proliferative DBR). In either case, early detection of these microstructural changes could allow for more immediate preventative action to be taken, mediated through drugs or other more invasive treatments. An expanded system would also potentially allow for examination of common skin diseases and screening for cancers, such as melanoma or basal cell carcinoma (BCC).^74^ Oral cancer and dental screening could also benefit patients, as oral caries and gingivitis alone pose a large financial burden and are extremely common.^75^

**Fig. 6:**
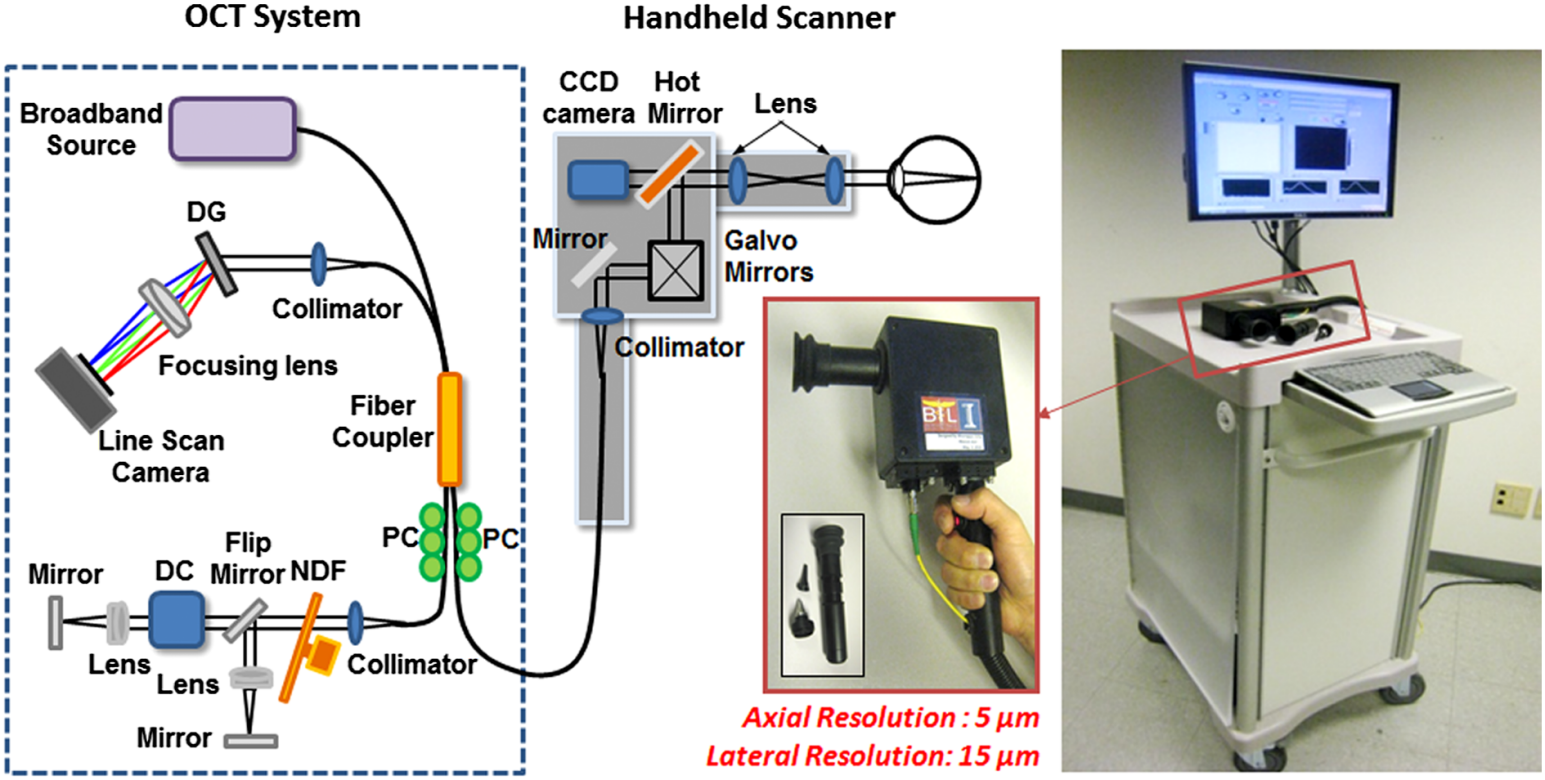
Schematic diagram and photograph of the first-generation primary care portable OCT system and handheld scanner. The cart included a broadband source, spectrometer, and additional optical hardware. The handheld scanner contained the sample optical path with galvanometer-based scanning mirrors. With interchangeable tip attachments, the physicians could easily access multiple tissue sites on a patient. Users were able to monitor both video and OCT images of the tissue and save images with a button mounted on the probe handle. Abbreviations: DG, diffraction grating; PC, polarization controller; DC, dispersion compensation materials; NDF, neutral density filter. Modified and reprinted with permission.^76^

This portable and multisite primary care imaging system and handheld probe, shown in Fig. 6, enabled optical characterization of these different sites with interchangeable attachments to the probe tip. An interchangeable adapter used a modified cutout that simulated the device head on a commercial otoscope, such that it would be possible to use standard otoscope tips for TM imaging. To observe the skin, oral cavity, or cornea, a metal standoff and eye cup were used, if applicable, which also had the same fitment. To image the retina, an additional adjustable lens and eyecup was developed, along with a flip mirror in the reference arm to have the proper (longer) pathlength. Suitable dispersion compensation to improve image quality was also added. This initial system and probe design lacked a way to visualize the real-time acquired video and OCT scans on the handheld probe, and had the optical patch cable exposed near the grip, both of which increased the difficulty of using the probe. Although the tradeoffs from the combination and implementation of this hardware were apparent during use, this system and handheld probe was the first of its kind.

**Fig. 7:**
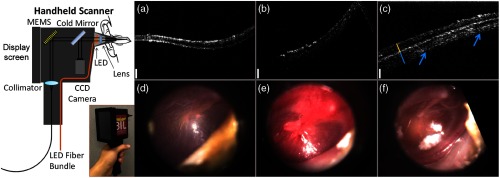
Redesigned first-generation primary care handheld OCT probe with representative data from pediatric human subjects with normal and infected ears. Left: Handheld unit was upgraded to include a MEMS-based scanning unit, new optics layout, and a small 5-in. LCD screen to display both surface video and cross-sectional OCT images during acquisition. (a–c) Representative cross-sectional OCT images of a normal ear, and ones with acute and chronic ear infections, respectively. (d–f) Representative corresponding digital otoscopy images. Modified and reprinted with permission.^79^

Later, the internal hardware of the handheld probe was redesigned to improve functionality in clinical settings, although the cart remained the same. A microelectromechanical system (MEMS) scanner replaced the pair of galvanometers to reduce the form factor of the handheld probe and provide more stable and adjustable scans. With smaller components, a color charge-coupled device (CCD) camera was also able to be included, although still at a fairly low 480 p resolution, for surface imaging. A 5-in. screen was added to the probe that allowed the user to visualize OCT data and the color surface image in real time. A commercial spectrometer and light source further improved the reliability of this system, which did not require regular maintenance, alignment, or cleaning to maintain performance, especially when moving the system to different clinical sites. This improved system was first used for research in the human eye at a local eye clinic,^77^^,^^78^ and later was used to investigate ear infections in adult subjects.^76^ Later, the Labview software was refined for a clinical environment, by incorporating larger and more accessible on-screen buttons, clearer display of relevant information, and simplified image and information display of only what was needed when imaging subjects. A separate version of the software was available to test and diagnose problems should they arise and provided information regarding system stability and base function. The improved usability of this system ultimately let the system operator better focus on interacting with the imaging subject. The system was then used at the outpatient otolaryngologist clinic to observe pediatric subjects and catalogued a normative database of different types and stages of ear infections. Notably, inflammatory changes in the TM and middle ear biofilms were regularly identified in human subjects with acute and chronic OM, respectively.^79^ Some representative images are shown in Fig. 7.

### 3.6 Second Generation: Design Improvements for Human Subject Imaging

**Fig. 8:**
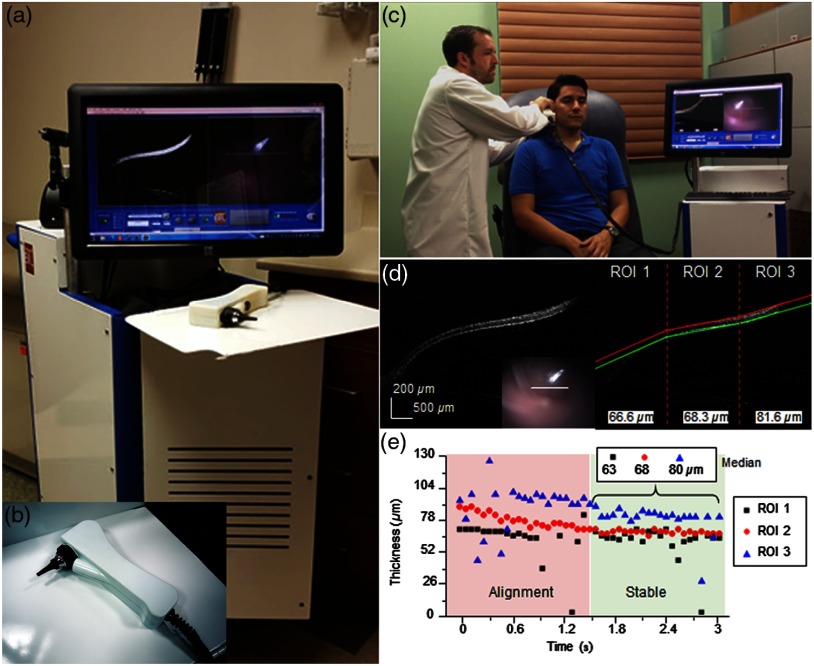
Second-generation primary care system and handheld probe. (a, b) Portable system and handheld probe in the primary care clinic. (c) System in use within our clinical imaging suite. (d) Automated segmentation and thickness analysis of an *in vivo* human TM. (e) Real-time thickness measurement tracking over time. Modified and reprinted with permission.^80^^,^^81^

For the second generation of handheld OCT probes for use in primary care settings, one goal was to refine the design of the handheld probe and portable cart for usability. A major consideration during design was in the portability of the system, as different research projects occurred at separate and distant outpatient or specialist clinics. To utilize existing automobile transportation, the height of the cart was limited, although the monitor mount had the ability to lower into the portable cart. Even with a reduced size, it is prudent for a research-based system to maintain access and flexibility for easy access for repair or to realign components, if necessary. Designing the optical layout and form factor of the handheld probe in digital CAD programs allowed for a rapid iterative design process that met design requirements without the need for a costly lens library and mounting equipment for testing. Similarly, through the use of a customized 3-D printed enclosure, the probe form factor was reduced by ∼30% over the previous iteration by designing only the needed support structures and outer enclosure, moving away from machined bulky metal fixtures. In total, these changes and modifications reduced the weight of the probe to less than 250 g and ensured that the optical alignment was stable through regular daily use and after transport to multiple imaging sites. The visualization screen on the handheld probe was also removed to reduce bulk, and other display and visualization platforms were explored, such as displays strapped to the operator’s forearms. While many integrated display options were available, all we identified were either limiting or did not have the desired appearance. For example, high-quality replacement screens for mobile devices are available to purchase, but driver boards and USB interfaces are rarely available. Other components were too compromised at that time, and had large bezels, or were too heavy to attach onto a handheld device. Finally, all electrical wiring and optical fiber were routed within a protective sheath that also provided some minor strain relief on the cabling, which was a crucial addition to protect the optical fiber from damage during use, storage, or transport. The finalized system and handheld probe are shown in Fig. 8.

During imaging sessions using these handheld probes, it was challenging for pediatric subjects to remain completely still and attentive. Subjects inadvertently moved through the imaging range of the probe, which was only several millimeters, even while sitting relatively still. While images could be stably acquired, motion was often erratic, making the collection of single scans by manipulating the on-screen “stop” and “record” buttons no longer possible, as commonly done in benchtop systems. Similarly, continuously recording all data was too intensive on computer resources. The operating software of this system was redesigned to provide improved saving functionality and file management by implementing a trigger system that allowed the operator to save a buffered queue of previously visualized on-screen images. Operation using this trigger would ensure any data acquired provided useful information for later analysis. Other improvements included an automated thickness measurement algorithm for rapid analysis of the TM,^80^ shown in Fig. 8, and a Google GLASS headset to visualize data^82^ since a screen was no longer included on this handheld. This system was later used to evaluate the *in vivo* viscosity of middle ear effusions, fluid that often accumulates behind the TM in subjects with OM.^81^ The success achieved in these various projects was in part due to the improved long-term reliability and ease of use of this system, as well as the reduced form factor and weight over the first-generation system. These qualities helped researchers to more efficiently explore various projects in both outpatient and surgical environments.

Additional studies were also conducted to further characterize the anatomical and biomechanical properties of the ear in the presence of OM. The discovery of biofilms adhered to the TM and identified with OCT in a previous study,^79^ and their continued implication in chronic OM,^83^ prompted further investigation into the role of biofilms in chronic infections. Biofilms dramatically promote bacterial resistance against antibiotics and immune system activity. Current clinical guidelines for treating OM do not take middle ear biofilms into consideration, as the standard otoscope does not provide any information regarding their presence or absence. The presence of biofilms in chronic OM may explain why tympanostomy tube (TT) placement surgery is most effective at resolving some of the more severe cases of chronic OM.^84^ TT placement surgery is a standard-of-care treatment^85^ for chronic OM that inserts a ventilation tube into the eardrum to allow for increased drainage and ventilation of the middle ear cavity, as well as to return normal hearing function. To study and investigate the pathogenesis of OM and the effects of standard-of-care treatment, including the surgical placement of TT, subjects with chronic OM were prospectively and longitudinally observed to assess for the presence or absence of middle-ear biofilms with treatment. This and other studies are currently ongoing to more fully characterize biofilm presence and structure during infection and treatment. If biofilms are shown to be a major driving factor in chronic OM, treatment guidelines may be updated after further study to take the presence of middle ear biofilms into account before prescribing antibiotics.

**Fig. 9:**
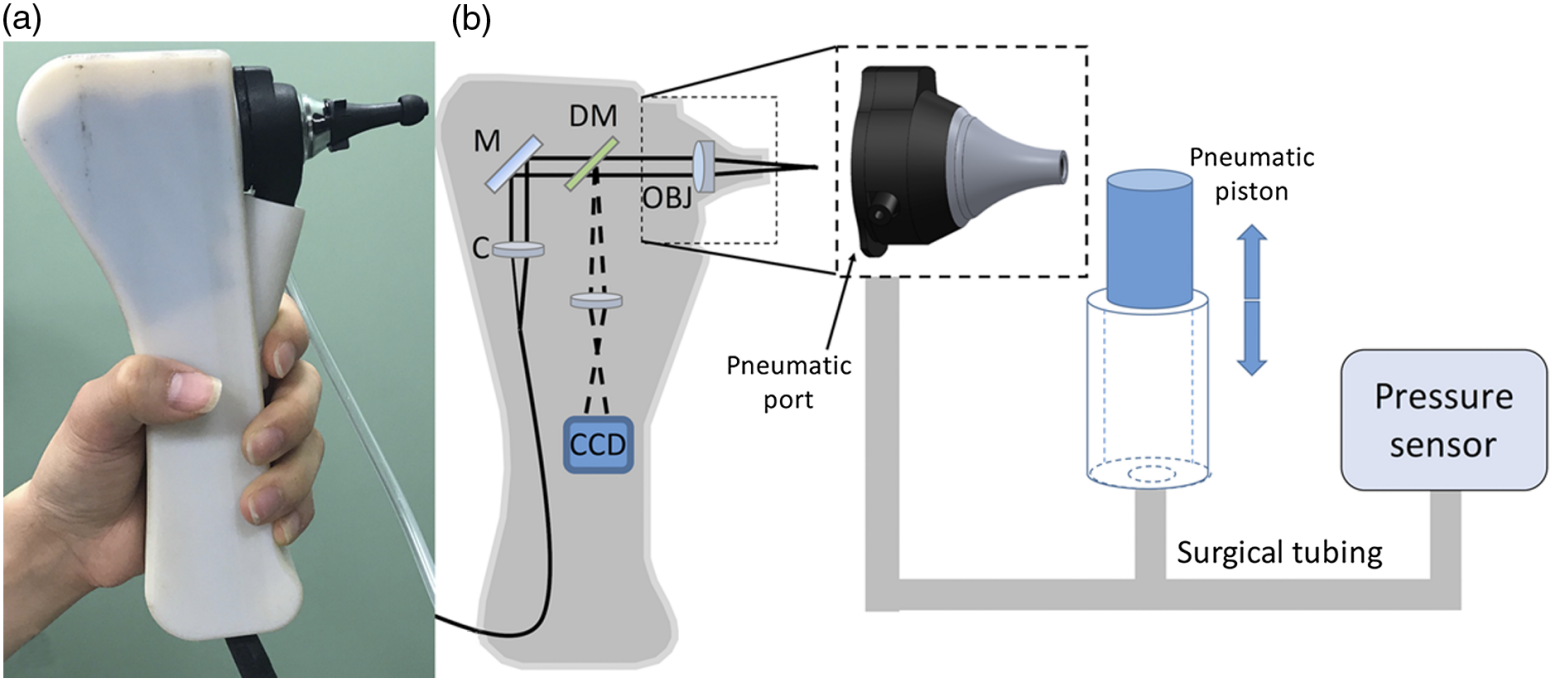
Second-generation primary care imaging handheld probe modified to accommodate pneumatic functionality. (a) A rubber ear-bud tip was added to the ear speculum to ensure a pressure seal within the ear canal, along with a pressure sealed probe tip. (b) System schematic showing an input port for the computer-controlled air pressure stimulus (dotted box). This fast axial-scanning LCI system replaced the two-dimensional (2-D) scanning MEMS-driven mirror to lower costs and capture fast dynamic transients of the TM following the pressure stimulus. Abbreviations: M, gold mirror; C, collimator; DM, dichroic mirror; OBJ, objective.^86^

In later iterations of this handheld probe design, pneumatic capabilities were added, as shown in Fig. 9, that allowed the first demonstration of high-resolution depth-resolved characterization of the TM using fast LCI axial scans with a pneumatic stimulus.^86^ Subsequently, lower-cost system components were implemented in attempts to reduce the overall cost, both in software^87^ and hardware.^88^

### 3.7 Third Generation: Tradeoffs in Complexity, Form Factor, and Cost

For the third generation of primary care systems, the focus centered on further reducing form factor and significantly reducing cost, to where primary care offices could potentially afford a shared system that retained the capabilities of previous generations. Two systems were constructed that utilized different OCT engines and construction materials to reduce cost and weight. Through this design process, powder-coated aluminum carts and side panels were found to be both functional and aesthetically appropriate against transport and daily use, especially when compared to carts created with aluminum frames and plastic panels. Wheel size was an important consideration, mainly to ensure that moving the system could be done smoothly and safely without fear of getting caught on gaps in elevators, or on door jambs, or even simply thick carpet. Larger 4- or 5-in.-diameter medical wheels were found to be optimal for our portable cart designs.

Realizing that repairs and alignment often required access to almost every part of the cart, a CAD program was used to design an esthetically pleasing exterior that could still offer unrestricted access to every component in the cart by removing side panels. The reference arm of the OCT system transitioned to a cage-based system, which greatly reduced the footprint needed to maintain its stability over time. On early handheld probes, commercial otoscope replacement bulbs were used to illuminate a fiber bundle within the probe tip. As technology evolved and became available, a customized light-emitting diode ring and MEMS-collimator alignment package (Advanced MEMS, San Francisco, California) was added to this probe, and thus the form factor was reduced even further.

**Fig. 10:**
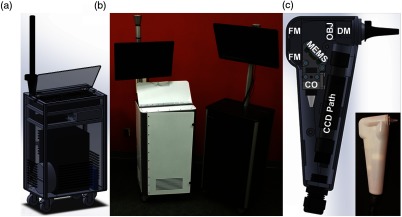
Third-generation primary care imaging portable system and handheld probe. (a) CAD schematic of cart layout and interior organization. (b) Portable systems testing different material choices (left: powder-coated metal frame and side panels; right: aluminum frame and plastic panels). (c) Schematic of handheld probe layout and final 3-D-printed enclosure. FM, fold mirror; CO, collimation optics; OBJ, objective lens; DM, dichroic mirror.

The overall cost of these systems was considered in relation to the choice of OCT engine and application. Spectral-domain OCT was often the preferred engine to maximize phase stability. New research opportunities utilizing swept-source OCT (SS-OCT) were investigated for imaging deeper into the middle ear cavity. While middle ear biofilms were previously found to be affixed to the TM, the middle ear mucosa was first shown to support biofilm growth.^89^ Having the capability to detect the presence and extent of biofilms simultaneously on the TM and on the mucosa at a potentially lower cost was a significant benefit. The tradeoffs explored between these two system designs allowed for a better understanding of the range of available commercial equipment at the time, tradeoffs between system design and form factor, and the capability for our intended application versus cost. Studies with these system designs are currently ongoing. When comparing the newer generation to the earlier generations, the portable cart form factor was reduced in size by nearly 40%, and the handheld probe was reduced by 10% to 20%, depending on grip shape. These later systems, shown in Fig. 10, eventually evolved into commercial prototypes.

### 3.8 Operating Within a Clinical Environment: Resources, Compliance, and Limitations

For many of the studies reviewed here, clinical imaging was performed in partnership with physicians at Carle Foundation Hospital in Urbana, Illinois. When clinical collaborations began in 2000, no dedicated staff or research space was available. However, as research needs grew, dedicated research space with access to the entire hospital through underground tunnels and loading elevators was constructed. This expansion also included research staff to assist in the recruiting and consenting of subjects, as well as assist with IRB compliance. Importantly, this space was used to store equipment, including different portable imaging carts, tools to maintain them, supplies to operate in a clinical environment, and a digital slide scanner to capture images of the corresponding histology. This space serves as an invaluable staging ground between university research labs and the hospital, especially before taking systems into a clinical or surgical environment. Similarly, the assistance from dedicated staff to recruit subjects that closely match recruitment profiles provided an immense operational benefit and were critical in gaining patient and physician trust and confidence in these imaging studies and with prototype research equipment.

One limitation in any clinical environment is the processing and handling of biological tissue, which is typically analyzed, documented, and stored in the pathology department, and cannot be transported offsite. As an initial solution, a relatively inexpensive microscope was built to digitize the histology slides from these clinical studies. These slides were used to correlate intraoperative OCT images against the current gold standard of H&E-stained histopathology. Later, a commercial digital slide scanner was purchased to enable rapid imaging and processing of entire histology slide sets. This system allowed high-resolution digital images of the entire set of histology slides for comparison, ensured that the original slides remained at the hospital, and enabled a more thorough correlation between histology and OCT images.

The use of OCT-needle biopsies was investigated in an attempt to improve breast cancer diagnosis rates and reduce the number of biopsy cores required for small, nonpalpable lesions. While needle biopsies are typically performed as outpatient procedures, it was not possible to develop manufacturing and sterilization procedures that would satisfy IRB and FDA requirements to use lab-built OCT needles and systems *in vivo*. Instead, OCT needle designs were developed and evaluated in freshly resected *ex vivo* human tumor masses within the operating room. This helped simulate insertion procedures into real tissue and provided a wealth of information on the structural features of fresh human breast tissue and tumors.^90^ This approach also provided an opportunity to develop logistics for the construction and handling of OCT needles in a clinical environment. Operationally, it was difficult to precisely correlate the location of the OCT image data with the corresponding histology, as it was often unknown whether a histology section would be analyzed along the same axis or orientation as our needle-based imaging. Similarly, the histological processing of the tissue dramatically changed its shape relative to the precise micron-scale positions of the needle-based measurements with OCT. While this initial study^91^ was essentially a “fine-needle” biopsy, later studies explored the development and use of “core needle” biopsies. A commercial OCT catheter-based system (LightLab/St. Jude Medical/Abbot Laboratories) was combined with a vacuum-assisted core-needle breast biopsy system (Hologic, Inc.) to enable imaging of both the tissue prior to and immediately after physical biopsy sampling.^92^ To move to *in vivo* human imaging and analysis, specialized protocols for sterilization were required for safety and IRB compliance of these modified probes. Other avenues of investigation were pursued.

### 3.9 Design Considerations for Handheld Probes

**Fig. 11:**
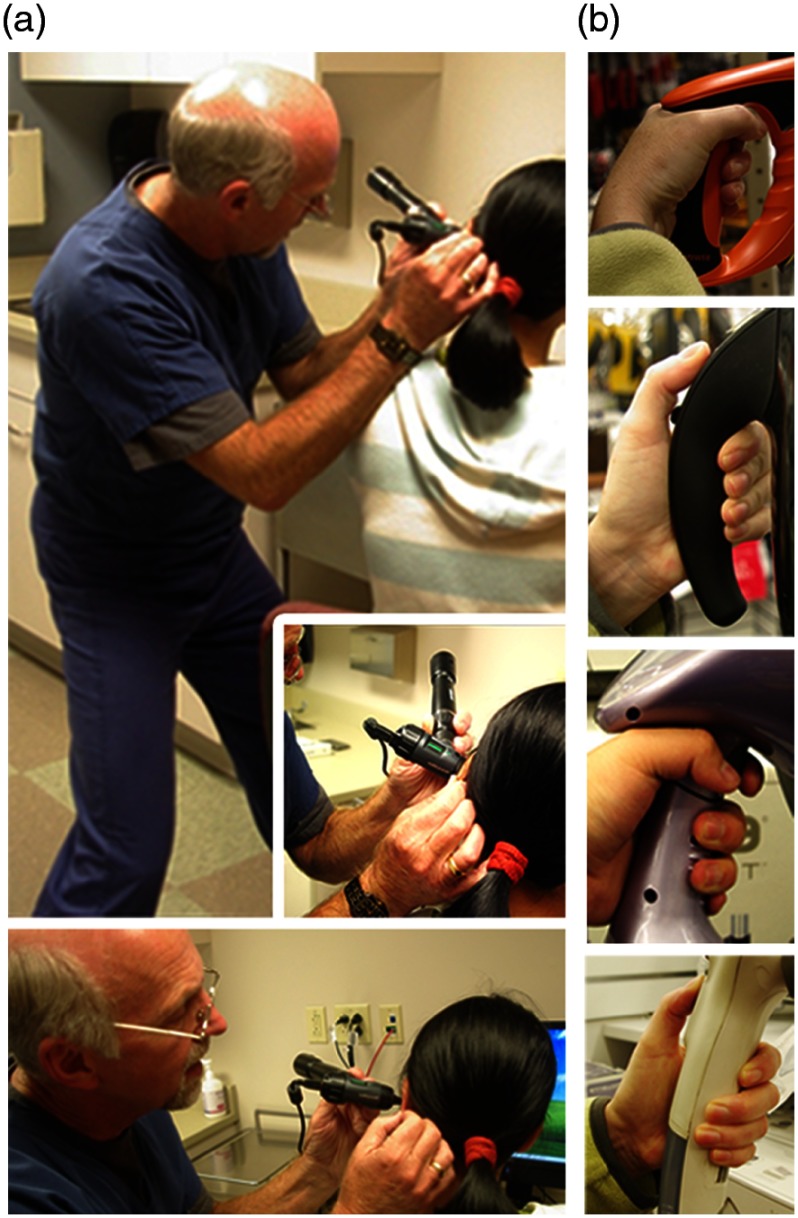
(a) Photos representing different handling techniques of an otoscope during use by an experienced ENT otolaryngologist and (b) different grip shapes, materials, and textures in various commercial products.

Ergonomic and user-focused designs are largely missing in many commercial products. A wealth of innovation, however, can be found in industrial design, which can be incorporated early in the development process to explore the usage and human interface between handheld probes and with actual users. Collaborating physicians were observed when using their own typical instruments during procedures, were questioned as to how and why certain tools were used, or held in a specific manner, and where their gaze was fixed during procedures to truly understand the design, intended use, and actual use of the instruments. With this information, the design of various handheld tools, appliances, and instruments used in different fields was then examined to find inspiration for handheld OCT probes. A range of product types and categories were investigated, including children’s toys, consumer healthcare products, hand tools for construction, and even kitchen tools, all of which had excellent ergonomics for their intended use. Representative images from this investigation are shown in Fig. 11. Mood and feel, often difficult to quantify, were also considered, focusing on how an individual interprets any subconscious cues by using, touching, and feeling a tool. The weighting of handheld tools and probes was explored to find a comfortable grip and balance using the same mass and location of optical components used in the handheld probe design, and for users with different hand sizes and anatomy.

Esthetics are also often overlooked in the development and design of portable carts and handheld probes. To facilitate clinical translation and use, systems intended for academic research in clinical environments should look like they belong in a clinical environment. The exterior appearance of the system should reflect the technical development efforts undoubtedly within. This equates to no visible loose wires or fitments, and no visible glue, tape, or other temporary measures for construction. As system designers, engineers are intimately familiar with their systems. Yet, clinical staff and especially imaging subjects are confronted with a completely new situation with a new instrument and new procedures, and carry their own perceptions, doubts, curiosity, and hesitation about any unproven research system or instrument. A unified appearance will help inspire confidence in clinical staff and imaging subjects that the system is safe, well-designed, and robust, and more importantly that it will provide new helpful data during operation. To reinforce this, even the color of portable carts and handheld probes should be modeled after current medical products, if possible.

**Fig. 12:**
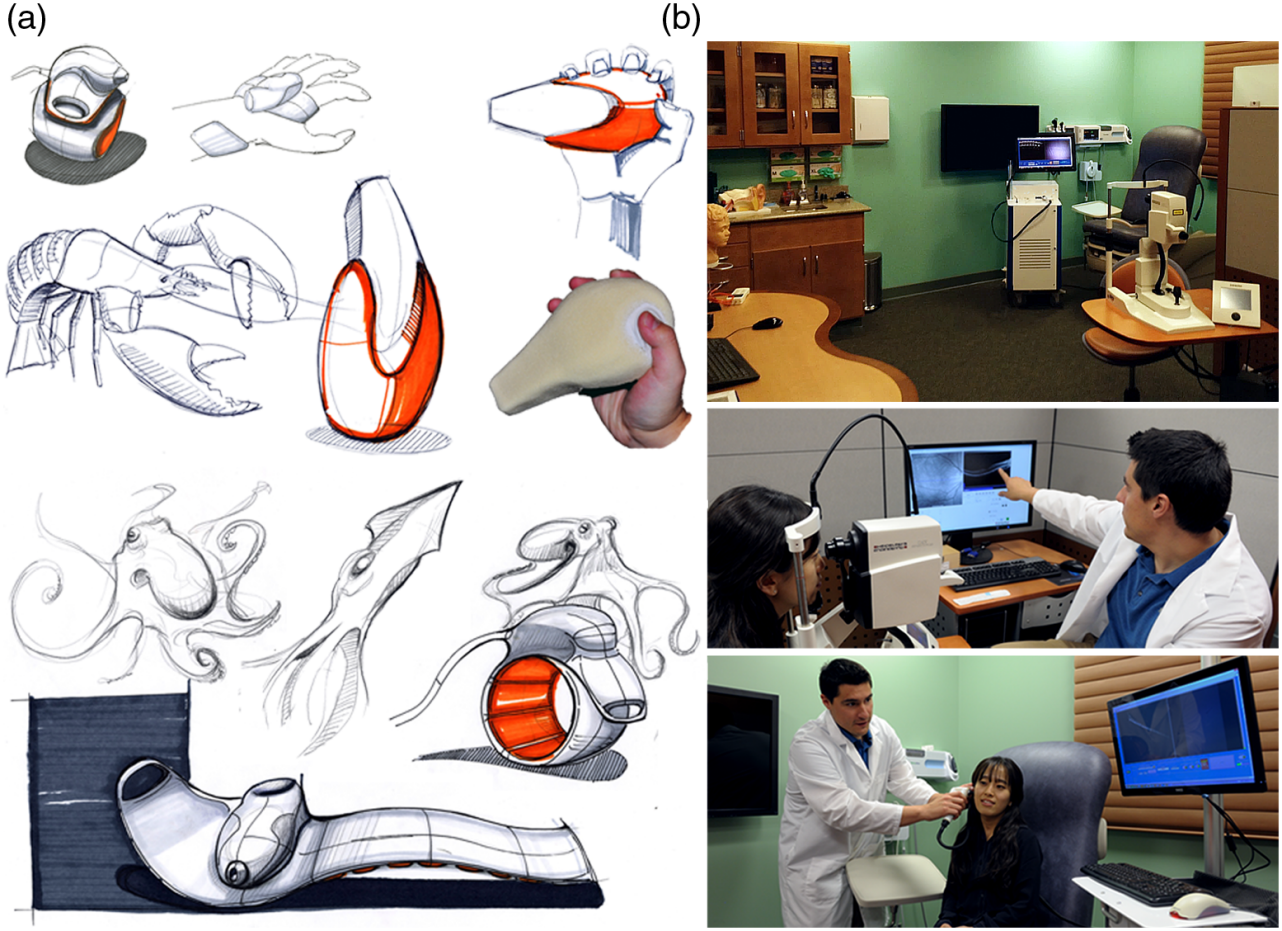
(a) Nature-inspired designs for handheld probes. Many future designs were considered for an ergonomic handheld probe. (b) A “doctor’s office of the future.” This lab space was redesigned for human subject imaging, with special attention given to color, layout, and function. The room not only included standard clinical equipment: otoscope, ophthalmoscope, blood pressure and pulse oximetry measurement systems, and sterile healthcare supplies, but also portable primary care imaging systems and teaching tools.

Working with an industrial design collaborator, nature-inspired designs for future handheld probes were created, with some concept designs shown in Fig. 12. In addition, other interaction and encounter spaces in the patient workflow were explored and redesigned to incorporate next-generation technologies such as OCT systems and handheld probes in primary care. A “Doctor’s office of the future” was designed where lab space was remodeled to support human subject imaging studies, and where human subject volunteers and visitors would feel as if they walked into a real clinical environment. Colors, textures, layout, furniture, and the available equipment in the room were concepts taken into account during the design and renovation of this space. 3-D plastic models and poster diagrams of the human ear, eye, teeth, and skin and a large wall-mounted display helped provide background information to subjects before their exam as well as an opportunity for self-teaching. The large display also allowed imaging results to be efficiently displayed, relayed, and explained to the subject postexamination. In addition, instead of relying on unclear or incorrect online sources, informational websites could be reviewed and explained transparently. This “Shared Care” concept would allow the imaging subject to feel more comfortable and involved in the clinical exam.

Finally, no single industry is able to provide the components needed to construct these custom-designed systems, and future system builders are encouraged to explore and creatively use components and parts from many suppliers, ranging from not only suppliers of optics, electronics, and hardware but also medical supply companies, plumbing fixture resellers, and the automobile industry, to name only a few.

### 3.10 Ensuring Stability During Handheld Probe Imaging

Apart from ergonomic and esthetic design choices of a handheld probe, ensuring stability of the subject and optical system during imaging is a challenging task for system designers and operators. The imaging depth of a typical spectral-domain OCT system is several millimeters, which requires accurate positioning of the subject within the focal plane of the handheld probe during image collection. With no external guide, operator and subject instability may prevent the acquisition of useful data. Desktop OCT systems typically employ a floating table, sample rest, and precisely controlled three-axis stage to ensure motion artifacts are minimized during image collection. For benchtop human subject imaging, fixed physical anchors have been effective, such as the ubiquitous chin rest and forehead bar used in retinal imaging.^93^ Although there are other involuntary ocular movements that occur, this mount reduces neck and body movements that would otherwise make imaging problematic. Similarly, other medical imaging modalities, such as MRI^94^ and CT,^95^ restrict subject motion and keep the subject relaxed by laying the subject horizontally, using blankets, and sometimes using restraints or anesthesia. Images can also be collected during periods of brief stability linked to either breathing^96^ or heart rate,^97^ as done in other imaging specialties.

While no solution can completely replace a stable system, there are several methods that reduce motion when imaging using handheld probes. Some specialized SS-OCT systems have recently expanded the interrogation depth from several millimeters to several centimeters using new MEMS-vertical-cavity surface-emitting laser technology.^98^ This helps to provide a wider (deeper) window for image collection, although it does not necessarily improve imaging stability. Some systems, such as buffered FDML systems, simply image at a faster rate,^99^ which can reduce the interframe movement if the tissue is in focus. Phase stability thresholds for specific applications can also be calculated.^100^ Most handheld probes are used in contact-mode, employing a precisely crafted standoff to assist the operator at keeping the positioning stable and focused on the tissue site. One common example of this is the ophthalmoscope eye cup that provides the system operator a guide for setting the proper distance from the eye, with finer adjustment done by hand for exact positioning. In some cases, the operator can use their hand and grip to stabilize the probe against the subject, or have the tip standoff anchored around the tissue itself and rely on gentle downward force to prevent movement.

To ensure stable image collection in a system, enabling some adjustability in the imaging range and working distance of the optical system is key. Similarly, a thorough knowledge of typical physiological dimensions and normative bounds of variation among the subject population can also help refine specialized standoff designs. Finally, any buttons or triggers on the probe used to initialize saving data must also be carefully chosen and placed. The trigger pressure, if too high, will cause minor movements that may inadvertently translate the imaging beam around the tissue surface and away from the originally intended interrogation site. Depending on the location of the button relative to the grip, especially if oriented directly along the imaging depth axis, similar translations may move the tissue in and out of the plane of focus.

This section provided a unique retelling of the evolution of intraoperative and primary care imaging systems based on the practical experience and insight from one research group. Many of the technical considerations for system performance, portability, and usability were considered iteratively and simultaneously alongside the operational norms of a clinical environment. Many other groups have also created robust probe and system designs that employ unique technical solutions to solve challenging issues in image acquisition, stability, and beam delivery for many other fields and applications. The next section highlights some of these systems. While many Stage 1 benchtop systems are the backbone of commercial applications of OCT, and other primarily benchtop OCT systems can be used in a handheld configuration with limited functionality, these systems will not be discussed here, as the focus is primarily on handheld OCT systems.

## 4 Stage 2 Systems: Biological Discovery Through Technical Innovation

There are a multitude of handheld systems that employ innovative technical solutions to provide a means to observe new biological phenomena. In this section, the varied landscape of Stage 2 handheld systems is surveyed, attempting to capture the breadth of handheld OCT systems and their technical innovations, as well as highlighting the various application subspecialties. While some commercial entities, research startups, and businesses are mentioned here, none are specifically endorsed by the authors of this paper. A thorough review and discussion of commercial OCT efforts is available elsewhere.^31^^,^^33^

### 4.1 Ophthalmology

Ophthalmology was one of the first research and application areas of OCT, since few other medical imaging modalities provided the needed resolution and noninvasive diagnostic capability to observe the retina and other ocular components. With the recent commercial release of software-based angiography processing in desktop ophthalmic systems, current research interest areas include viewing retinal cells beyond the rods and cones,^101^ wider fields of view,^102^ and high-speed imaging.^103^ Many different systems have been constructed toward achieving one or several of these aims by both research and commercial interests.

**Fig. 13:**
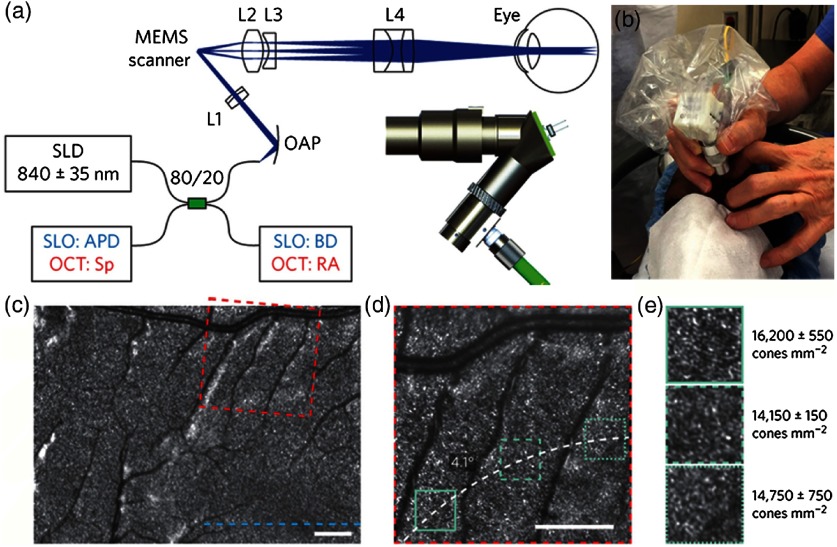
Ultracompact SLO/OCT high-resolution handheld probe with widefield images assembled through mosaicking. (a) Optical design, schematic, and 3-D rendering of ultracompact handheld probe. SLD, superluminescent diode; APD, avalanche photodiode; Sp, spectrometer; BD, beam dump; RA, reference arm; OAP, reflective collimator (off-axes parabolic mirror); L1–L4, lenses. Blue and red text in the schematic corresponds to components used for imaging in the SLO or OCT modes, respectively. (b) Probe in use in a pediatric human subject. (c) SLO image near fovea. (d) Zoom of (c) (dotted red) to parafoveal receptors. (e) Zoom of (d) (dotted teal), with density calculations in several regions across (d). Modified and reprinted with permission.^110^

One excellent example of the evolution of a handheld design in principle and execution is demonstrated in a combined scanning laser ophthalmoscope (SLO)-OCT handheld retinal system. SLOs are typically used to collect high-speed reflectivity maps of the retina^104^^,^^105^ and when used with fluorescein dyes can visualize vasculature.^106^ When combined with OCT, a fundus image can be quickly generated along with cross-sectional depth scans.^107^ Multiple reference designs, CAD drawings, and optical designs have been provided that demonstrate a cohesive effort to produce handheld OCT probes. The original design uses what appears to be a combination of off-the-shelf and custom fabricated aluminum parts to allow for a relatively compact unit.^108^ A second-generation version^109^ further reduced the form factor and increased the optical capabilities of the first. As shown in Fig. 13, a third-generation version produced high-resolution images of the retina in a compact handheld,^110^ shown in pediatric subjects, who are typically more difficult to image than adults. When compared to the first version, its size was reduced by nearly 40× in total volume, yet the probe was still able to produce images of similar quality with a lateral resolution of ∼7  μm in a model eye, and of sufficient capability to detect the varying densities of parafoveal rods and cone cells in the retina.

**Fig. 14:**
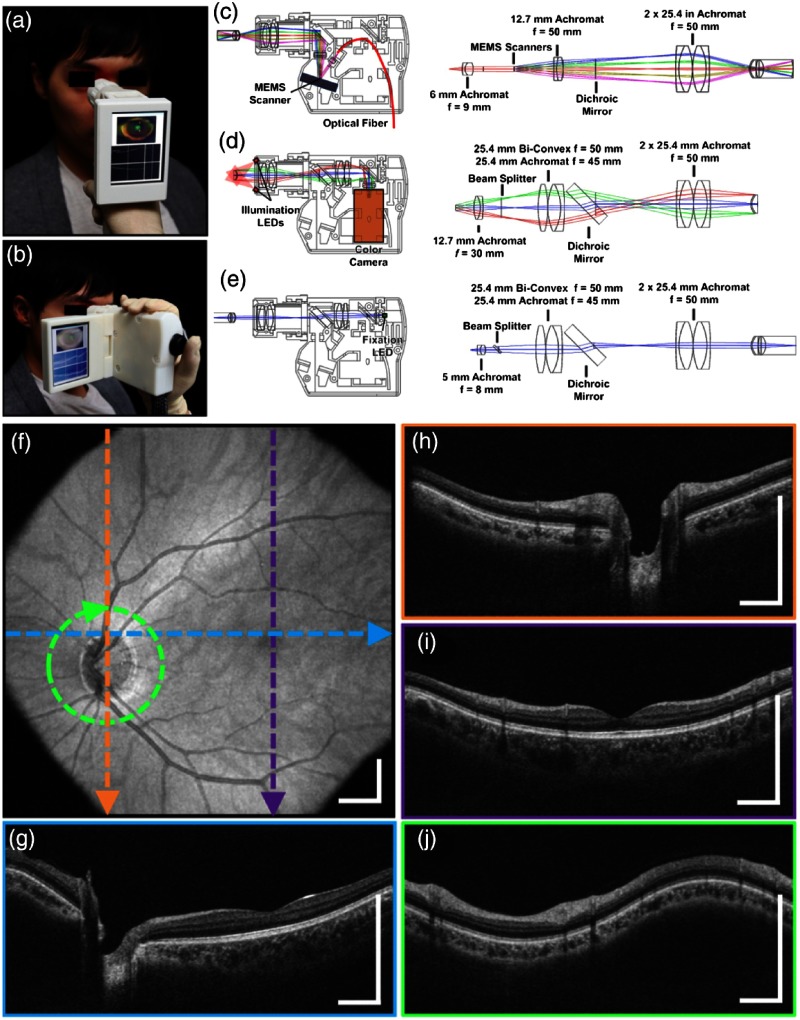
Handheld MEMS-based OCT probe. Demonstration of different grip styles including (a) “power grip” and (b) “camcorder” styles. (c–e) Optical layouts of (c) OCT 1060 nm optical path, (d) iris camera visible optical path, and (e) fixation target visible optical path. (f–j) Motion-corrected widefield volume; (f) *en face* OCT fundus image. (g–j) Color-indicated OCT scans from various regions in the retina. Modified and reprinted with permission.^111^

Another standout handheld design provided high-speed and widefield observation of the retina in a flexible package.^111^ This system allowed for efficient screening of retinal pathologies in patients in a clinical environment, given its simplified data collection and fast imaging rate to reduce motion artifiacts. This handheld had an adjustable grip orientation and provided scans of the retina of up to $10\times 10\;{\mathrm{mm}}^{2}$ using a 350-kHz A-line rate VCSEL source and photodiode. The handheld screen displayed both a representation of the fundus/iris view and the OCT scan. An optical design schematic illustrates the three optical design paths within the handheld: OCT, iris/fundus camera, and fixation target (Fig. 14).^111^ Robust handheld systems such as these allow for enhanced screening for ocular trauma or early retinal disease in settings outside of typical clinical environments.

**Fig. 15:**
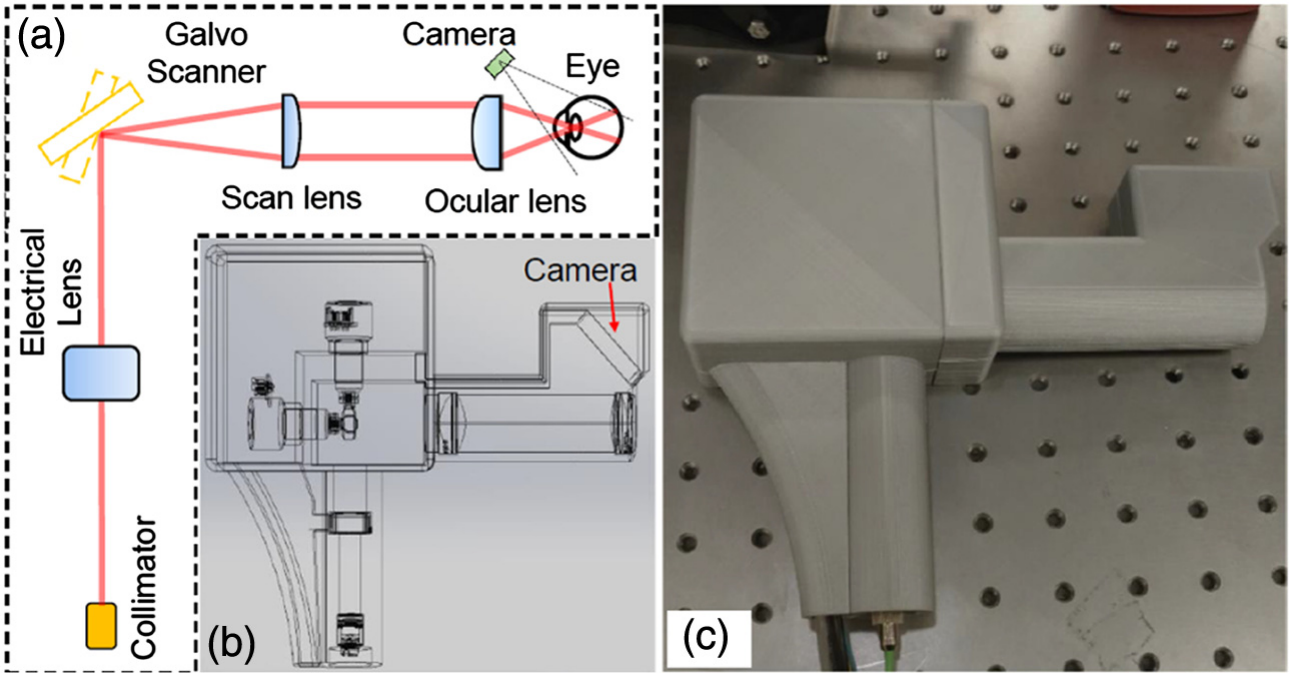
Handheld OCT angiography probe with automated tunable lens focus adjustment. (a) Schematic of the handheld OCTA probe. (b) 3-D perspective view of the probe including all the optical components and the designed plastic case for mounting and holding. (c) Photograph of the handheld OCTA probe with 3-D-printed enclosure. Reprinted with permission.^114^

Angiography is a major focus in clinical imaging of the retina,^23^^,^^24^^,^^112^^,^^113^ since it provides a view of the vasculature without added dyes or contrast agents. With phase stable OCT data, functional time-resolved changes from blood cells in the microvasculature of the retina can be calculated to create an *en face* angiogram without the need for any injected contrast or fluorescent dye.^23^ This is technically difficult to achieve in a handheld, since these techniques are typically limited by the phase stability of the optical system and the optical fiber connected to the handheld probe, as well as the inherent added motion of the user holding the probe and the subject being imaged. One handheld probe has been able to overcome these challenges, notably without the use of mydriatic (dilation) drops, to produce volumetric angiograms of subjects (Fig. 15).^114^ This system utilized a fast-refocusing tunable lens to quickly lock onto the retina and postacquisition B-scan alignment to correct for motion artifacts from the operator and subject. The sensitivity of the system for imaging capillaries in the retina approaches that of mounted commercial systems and can provide a vessel density map of the retina without the use of any injected contrast agents. This information is useful for clinicians to discern pathological vessel growth in subjects with DBR or other diseases.

**Fig. 16:**
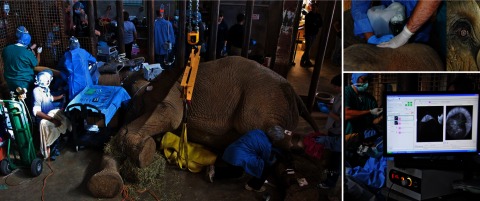
Surgical removal of cataract from C’sar, an African elephant at the North Carolina Zoo. The handheld *Envisu R2300* OCT scanner from Bioptigen was used to guide surgeons on this delicate surgery, dramatically improving the quality of life for this patient. Image courtesy of Eric Swanson, *OCT News*, and Eric Buckland, *Leica* (Bioptigen).^115^

Portable and handheld system designs have the capability to bring OCT to atypical situations that require high-resolution imaging and where large benchtop setups are prohibitive. Very often, it is more practical and sometimes necessary to bring the imaging system to the subject, rather than having the subject approach the benchtop imaging system. In one unique case, an elephant was nearly blind and required cataract removal surgery to improve its quality of life. With assistance from a portable OCT system and handheld probe, surgeons were able to remove the cataracts and restore partial vision to this animal,^115^ although a synthetic lens implantation was not possible given existing damage to ocular tissue. The surgical team is shown with the anesthetized animal in Fig. 16.

### 4.2 Endoscopy

There are many target tissue sites that can be observed within the human body via an endoscope, especially when enhanced with OCT.^116^ Probes with a long distal tip have been used to reach deep into the larynx or the sinuses to allow visualization of these regions. OCT catheters can be directed through the many branches of the cardiovascular system^117^ or through the gastrointestinal tract,^118^ and needle-based probes can enable an internal view of solid tissues.^119^ Ultimately, endoscopic systems allow a user to view tissue within the subject that would otherwise not be possible. These systems rely on their shape or rigidity to navigate the potentially tortuous pathways inside the body, and a rotational or forward scanning mechanism to visualize internal structures of interest. Many of these systems attempt to provide an earlier or improved detection rate of cancer, ameliorate the poor survival rate of some cancers,^120^ to examine surgical margins,^121^ assess for the presence of plaques and weakened arterial structure,^118^ or provide contrast or functionality not available with more traditional techniques, such as Doppler OCT.^122^

**Fig. 17:**
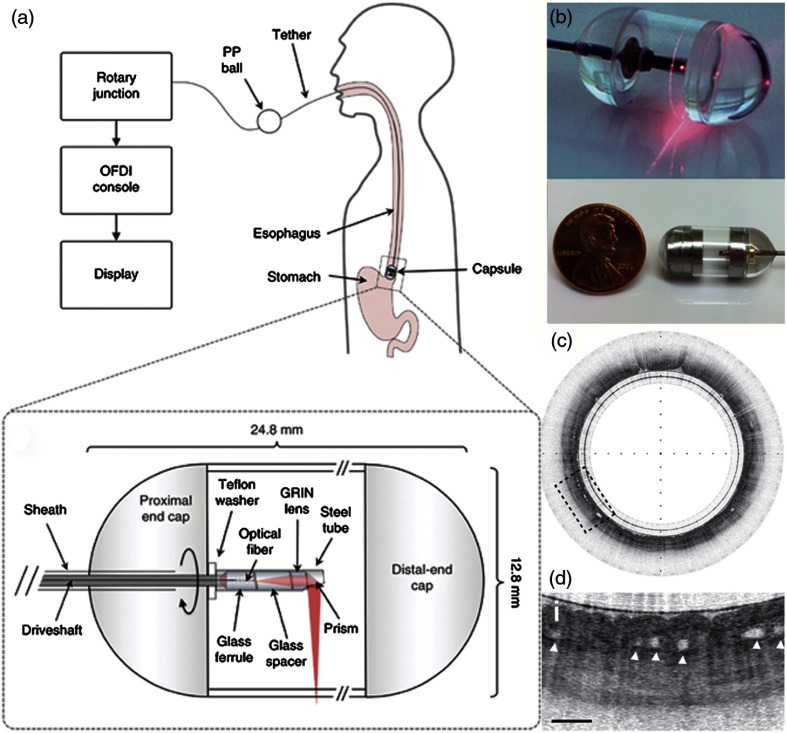
Tethered capsule OCT endomicroscopy. (a) Overview of the device. The tether allows retrieval of the capsule, which also contains the drive shaft and optical fiber, after being swallowed through the esophagus and passing briefly into the stomach of the subject. Inset: schematic of the capsule. (b) Close-up of capsule with scanning mechanism active and positioned adjacent to a US penny for scale. (c) *In vivo* tethered capsule endomicroscopy image obtained from a patient with histopathologically confirmed Barrett’s esophagus. (d) Threefold (3×) expanded view showing an irregular luminal surface, heterogeneous backscattering, and glands within the mucosa (arrowheads). Tick marks in (c) represent 1 mm. Scale bars represent 0.5 mm. Reprinted with permission from Macmillan Publishers Ltd.^123^

One such example relies on a tethered pill-like capsule that is used as an OCT probe to produce high-resolution images of the esophagus in order to screen for and detect Barrett’s esophagus.^123^ Initial versions of this system were developed and translated through an iterative process to produce the version currently found in the literature, as shown in Fig. 17. This system was designed for regular clinical use, which took into account the additional considerations and restrictions needed in a clinical environment and to use the device *in vivo*, such as patient safety and comfort, probe design, and rapid speed of imaging, to name only a few. Many of these aspects are detailed in prior publications.^118^^,^^124^ The tethered capsule design provided a means to access part of the stomach and the entire esophagus with OCT, and this system is now being used in outpatient settings.^124^ In the future, it may be possible to diagnose pathological esophageal conditions using high-resolution OCT images without the need for biopsy, and do so without the need for specialized healthcare resources or personnel.

**Fig. 18:**
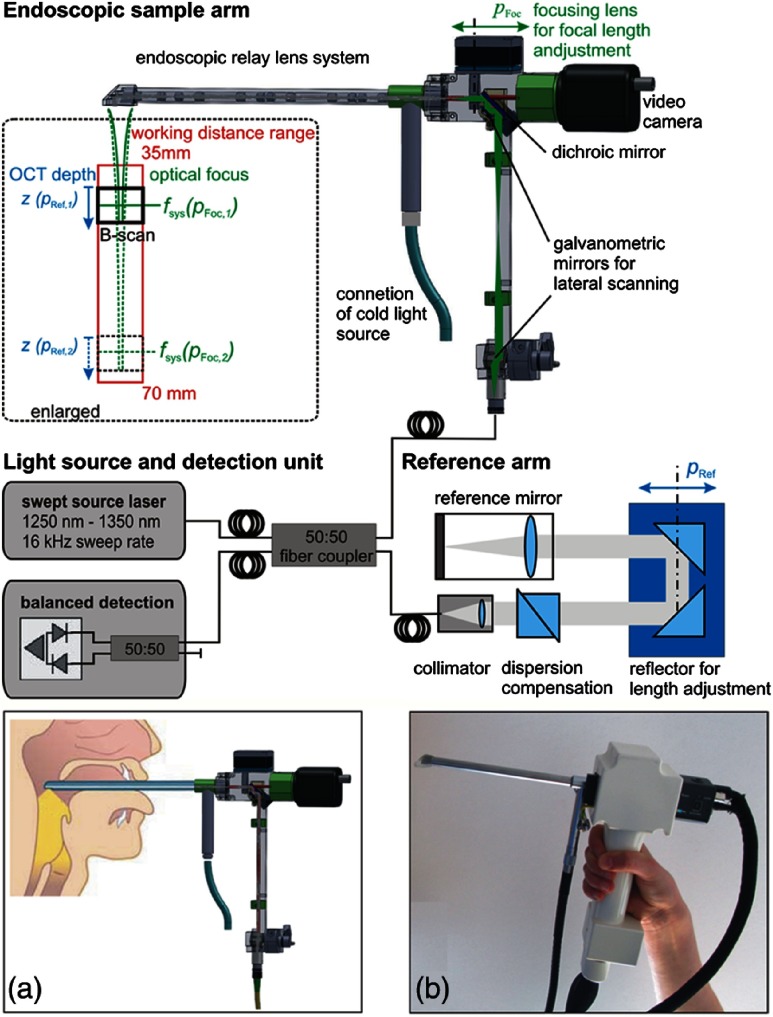
Schematic of a handheld rigid laryngoscope-based probe and OCT system. The working distance of the OCT-laryngoscope optical focus can be adjusted by the automated focusing lens, indicated in green, and by the OCT reference arm path-length, indicated in blue. (a) Handheld probe and interrogation area near vocal folds. (b) Photo of handheld probe. Reprinted with permission.^126^

Rigid endoscopes are also favored when examining the small airway opening or larynx.^118^^,^^125^ One laryngoscope system, shown in Fig. 18, employs a fast and automated focus adjustment unit in the body of the handheld and reference arm, such that the system can interrogate tissue in and around the vocal folds without adjusting the reference arm in the OCT imaging system.^126^ This is particularly useful to dynamically observe phonating vocal chords, and one excellent example of the handheld probe design ensuring stability during imaging. A swept-source-based OCT system also improves the imaging depth over a spectral-domain-based system.^35^ In total, this system provides quantitative structural and functional information into mucosal behavior during phonation, expanding the examination capability for other voice disorders.

**Fig. 19:**
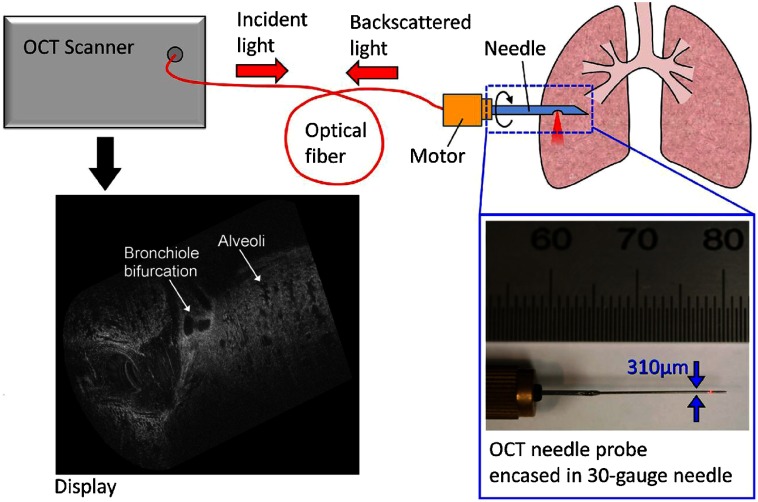
Schematic of a handheld OCT needle probe system with a 30-gauge needle. Display shows the 3-D visualization of a fetile lamb lung showing alveoli and bronchiole bifurcations. Length of cylindrical scan is 2 mm. Reprinted with permission.^132^

Needle-based endoscopes or probes allow a view into thick or solid tissue that is typically not possible with traditional imaging systems. There are numerous OCT-based needle probes that allow investigation of the prostate,^127^ renal tissue,^128^ deep brain imaging,^129^ guidance of epidural anesthesia injections,^130^ breast tissue,^90^^,^^92^ and the examination of the birefringent properties of breast tumors.^131^ One system enabled the investigation of dynamically moving alveoli in an animal model,^132^ as shown in Fig. 19.

**Fig. 20:**
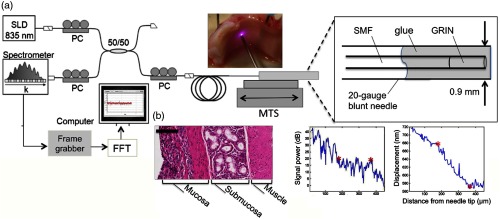
Tissue boundary detection using OCT-elastography in a handheld needle probe. (a) Schematic of forward facing needle probe and OCT system. Abbreviations: SLD, superluminescent diode; PC, polarization controller; MTS, motorized translation stage; SMF, single-mode fiber; GRIN, gradient-index fiber. (b) Boundary detection in *ex vivo* porcine tracheal wall with comparative histology. Scale bar represents 100  μm. Reprinted with permission.^138^

Functional OCT imaging provides information about a sample beyond structural morphology, such as in elastography. Elastography of skin and other soft tissue is performed by the modulation of pressure (force) applied to a sample, and based on the dynamic response, the biomechanical properties of the sample can be extracted. Although there are fairly rigid experimental requirements to perform this work, many tissue types have been explored and characterized.^133^^–^^136^ One example is through the use of a commercial probe that was adapted to detect the biomechanical properties of human skin with aging by observing elasticity and scattering metrics, and was performed *in vivo* on human subjects.^137^ Still, employing elastography techniques in a handheld probe-based system adds considerable difficulty, as the exact experimental and boundary conditions cannot always be guaranteed. Figure 20 shows one system that provides a method to detect tissue interfaces and was demonstrated in *ex vivo* tissue.^138^ A forward-scanning needle probe was used to detect tissue interfaces with this technique.

### 4.3 Cardiology

In cardiology applications, the challenging clinical environment and tortuous access to the human heart places stringent requirements on OCT probes and systems. While research in cardiology began soon after the inception of OCT,^139^^,^^140^ and has continued since,^141^ intravascular ultrasound (IVUS) still dominates this clinical application. For example, OCT-guided stent placement and IVUS have been shown to have comparable procedure rates despite the improved resolution of OCT.^142^^,^^143^ However, most physicians appreciate the improved image features and resolution that OCT provides, which is ∼10× the resolution of a standard IVUS image.^144^ With further research demonstrating, the utility of OCT over IVUS,^145^ systems demonstrating improved operating speed and ease of use, and especially a reduction in cost, OCT will likely continue to be impactful in cardiology. Most systems used in cardiology applications are catheter based^146^ and used primarily for the identification of plaques in the coronary arteries,^147^ evidence of atherosclerosis,^148^ and for stent placement.^147^

### 4.4 Dentistry and Dermatology

**Fig. 21:**
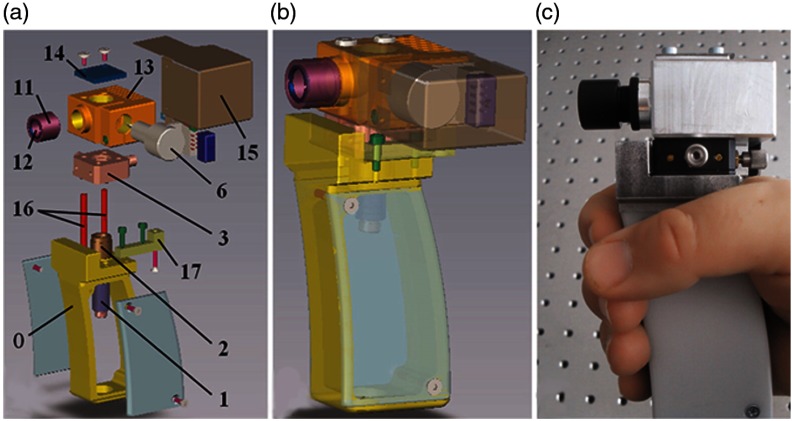
Handheld OCT scanner with a one-dimensional galvanometer-based scanner, suitable for small-scale production. (a, b) Exploded view (itemized) and transparent CAD design of the assembly. 1–17: optical components (see citation). (c) Completed handheld probe. Reprinted with permission.^154^

There are many applications in dentistry where OCT may be useful in the diagnosis of periodontal diseases,^149^ as well as for oral cancer,^150^^,^^151^ and in observing the effectiveness of various therapies for preventing enamel loss^152^ or for restoration,^153^ among many others. One group in particular prepared a highly detailed guide on engineering efforts to design, construct, and test several iterations of a handheld probe for general dental applications,^154^^,^^155^ reviewing design requirements such as material choices and cost. Figure 21 shows the compact and handheld “final prototype” OCT probe developed through this process. Commercial OCT probes for dentistry have been released^156^ and are beginning to find their way into clinical use.

When applied to the skin, the largest organ in the human body, OCT is often used to screen for melanomas or carcinomas,^157^^,^^158^ or to characterize more common dermatology lesions^159^ such as rashes, mites/parasites, inflammatory diseases,^160^ even acne.^161^ Handheld systems in this specialty need to be able to provide as deep imaging as possible to visualize the full-thickness structures of skin, along with other OCT variants that help to differentiate these conditions, such as Doppler or vascular imaging, to measure the local density of vessels.^162^ One handheld Gabor-domain OCM probe was able to provide cellular-level resolution in *en face* OCM images of skin.^163^ Special scan patterns were developed for this system to provide distortion-free imaging using an MEMS-scanner without postprocessing at 2  μm resolution (axial and lateral). Other commercial systems utilize multibeam and multifocus techniques to achieve a wide field of view and an extended axial imaging range.^158^^,^^164^

### 4.5 Surgical OCT-Enhanced Tools

**Fig. 22:**
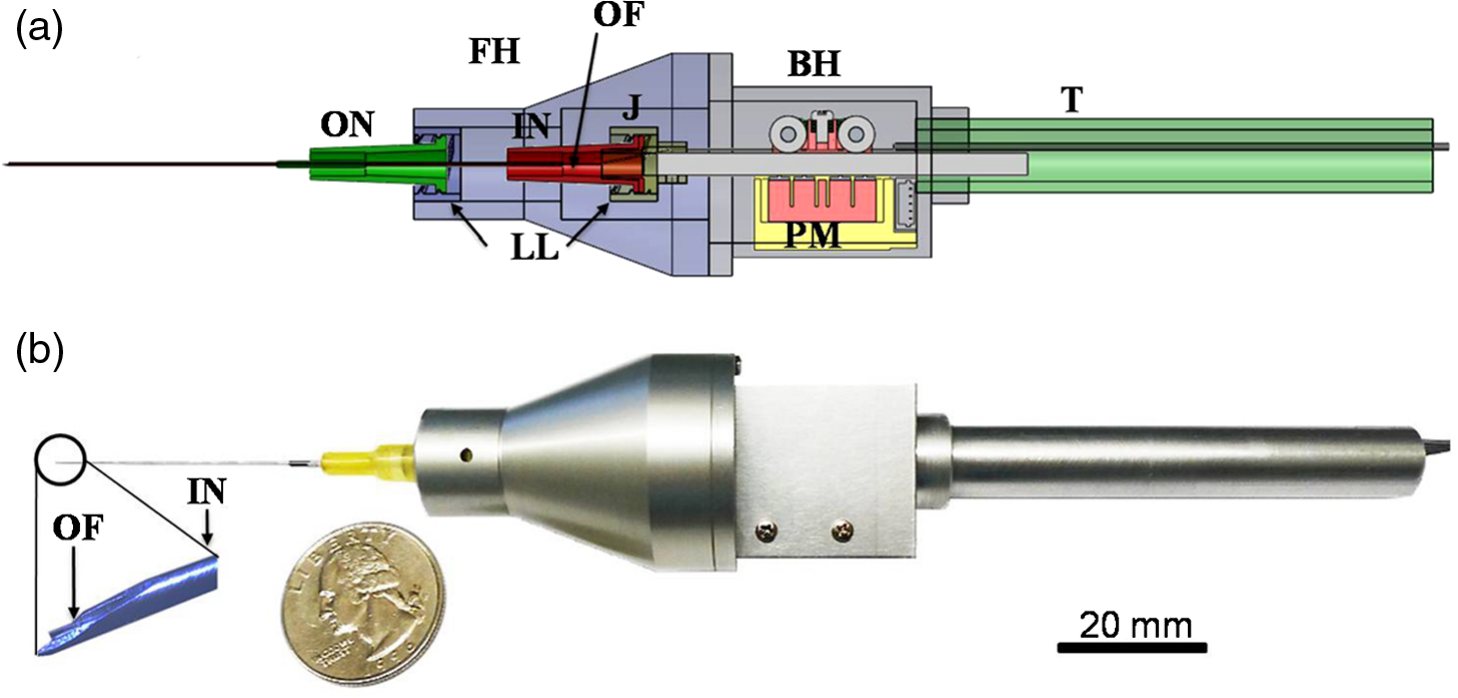
SMART handheld. (a) CAD design cross section. Abbreviations: FH, front holder; BH, back holder; J, joint; T, tail; ON, outer needle; IN, inner needle; PM, piezoelectric motor; LL, luer-lock combination; OF, optical fiber. (b) Completed probe, compared to a quarter (US currency). Reprinted with permission.^167^

Many handheld tools have been developed to fulfill a specific functional purpose and provide feedback during delicate surgical procedures.^165^ Many other surgical tools exist but in a benchtop configuration, depending on clinical needs and workflow. For example, a set of OCT-enhanced surgical microforceps can provide precise depth-positioning for removing thin membranes in retinal surgeries.^166^ Another vitreoretinal needle-based surgical tool was combined with a miniaturized OCT probe, shown in Fig. 22. The probe utilized a piezoelectric motor to stabilize this vitreoretinal tool in one direction for microsurgery, dampening the natural swaying motions in the hand from 0 to 15 Hz. Dubbed a SMART (smart micromanipulation aided robotic-surgery tool) handheld, stabilization results were verified on a live embryonic animal model. Tools like these will improve a user’s free-hand accuracy when manipulating tissue.

**Fig. 23:**
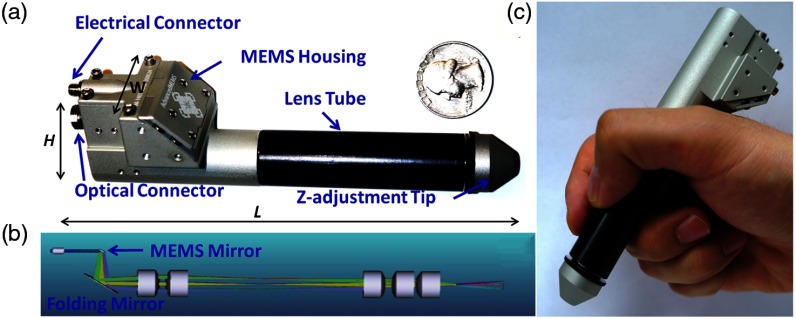
Intraoperative MEMS-based handheld Doppler OCT probe to observe microvascular anastomosis. (a) CAD design of handheld probe compared to US currency (quarter). (b) Interior optical design within probe. (c) Handheld probe in hand. Reprinted with permission.^168^

Another system, shown in Fig. 23, incorporates a compact MEMS-based handheld that can provide Doppler OCT images of vessels to observe successfully reconnected microvascular anastomosis, or the connection of fine vessels and structures during surgery. These and similar systems^169^ utilize the precise positioning OCT or LCI provides to precisely manipulate tissue in surgical procedures, which will likely improve patient outcomes.

## 5 Multimodal Systems: Overcoming Inherent Contrast Limitations in OCT

As discussed briefly, handheld OCT systems have enabled real-time high-resolution cross-sectional imaging from compact units for numerous clinical applications. Despite the ability of OCT to generate images based on the microscopic morphological features of tissue, the contrast in OCT is based largely on the inherent optical scattering properties of the tissue.^170^ The lack of chemical or molecular specificity brings additional challenges to the interpretation of OCT images, especially when classifying normal and pathological tissues solely based on morphology.^171^ In addition to contrast limitations, OCT has limited imaging depth due to multiple scattering, and constrained axial or lateral resolution when specialized imaging configurations are not used. To overcome these inherent limitations, many multimodal benchtop systems integrate OCT with another optical imaging modality. Nonlinear and multiphoton microscopy,^60^^,^^172^ fluorescence, Raman spectroscopy (RS), and photoacoustic imaging have been integrated with OCT to leverage the strengths of each modality.^32^ In this section, recent advances in the development of multimodal handheld probes are highlighted.

**Fig. 24:**
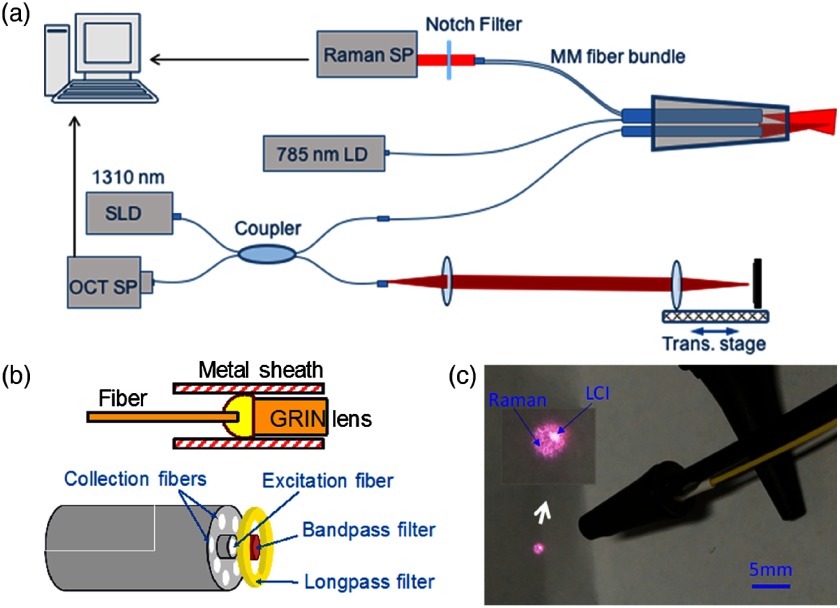
Multimodal handheld probe for sampling ear infections. (a) Schematic setup of the multimodal RS-LCI probe. (b) Schematic of the LCI (top) and RS (bottom) components. (c) Photograph of the fiber-based RS-LCI probe encapsulated into an ear speculum. The inset shows magnified view of both the RS and LCI beam. Abbreviations: SP, spectrometer; LD, laser diode; SLD, superluminescent diode; and MM, multimode. Reprinted with permission.^178^

RS provides specific biochemical analysis on a single-cell level, based on the optical vibrations from inelastic light scattering phenomena.^173^ However, its practical feasibility for clinical use is challenging due to the inherently weak Raman scattering and limited spatial information when using power densities that are safe for *in vivo* use.^174^ The integration of RS with OCT (RS-OCT) has allowed both morphological and biochemical characterization of the tissue in real-time. Several handheld RS-OCT probes have implemented a common sample arm with independent detection schemes to characterize skin cancer lesions^175^ and oral tissue.^176^ A common detector system was also presented for retina and skin imaging^177^ by incorporating a spectrograph for detection of both OCT and RS signals. Recently, a fiber-based handheld RS-LCI probe, shown in Fig. 24, has been developed.^178^ The multimodal system was able to differentiate microbial pathogens of infected ear fluid using RS, while LCI provided both depth-resolved microstructure as well as guidance of the RS beam for coregistration. In the future, it may be possible to simultaneously detect a middle ear biofilm and/or fluid, as well as identify the specific strains of bacteria that are present.

**Fig. 25:**
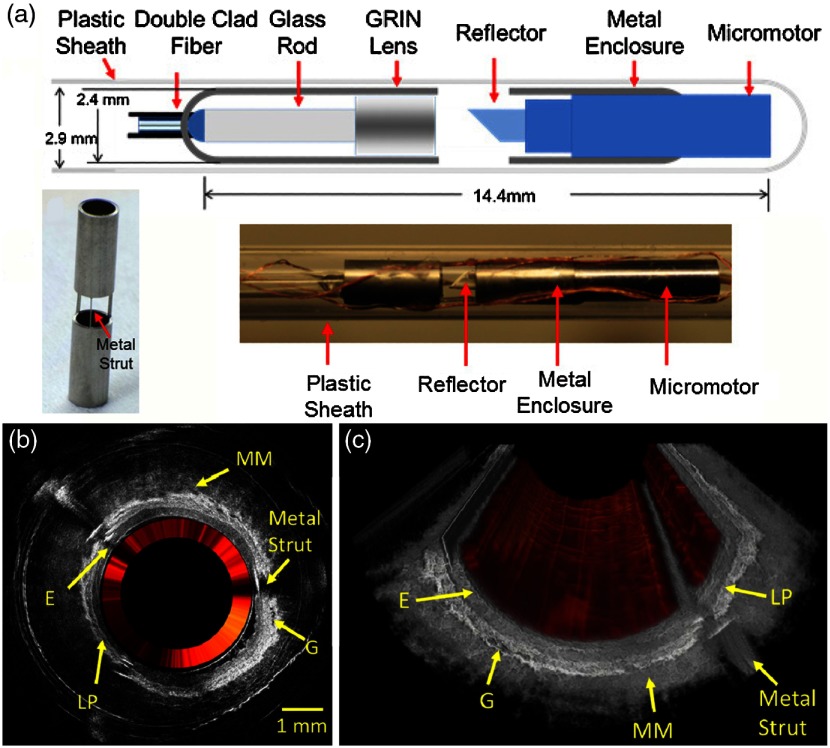
Combined OCT and FI probe schematic and results. (a) Schematic of distal end of the dual-mode endoscope design. A precision-cut metal enclosure (outer diam.: 2.4 mm) shows struts for minimal beam blockage (<5%). A photograph of the constructed endoscope including the transparent sheath is shown below (outer diam.: 2.9 mm). (b) Representative 2-D cross-sectional OCT image and (c) 3-D volume of *ex vivo* rabbit esophagus (gray) with the overlaid inner annulus (red) of the fluorescence intensity. The normal layered structures of the esophagus can be clearly visualized, including the epithelium (E), lamina propria (LP), muscularis mucosa (MM), and glands (G). Reprinted with permission.^182^

Fluorescence imaging (FI) is another optical technique that can be used to generate biomolecular information from tissue. Combined OCT and FI systems have been demonstrated in various imaging probe configurations. Catheter-based probes^179^^,^^180^ have been developed to investigate the cellular microstructure in areas with inflammation caused by atherosclerosis, and similarly in endoscopes, to identify colon cancer.^181^ One endoscopic system simultaneously delivers and detects OCT and FI signals using a custom dual-clad fiber,^182^ shown in an all-fiber-optic platform in Fig. 25. By correlating signals from both OCT and fluorescence, a user can simultaneously visualize the vasculature (using FI) and deeper subsurface structures, such as layered tissues and glands (using OCT).

**Fig. 26:**
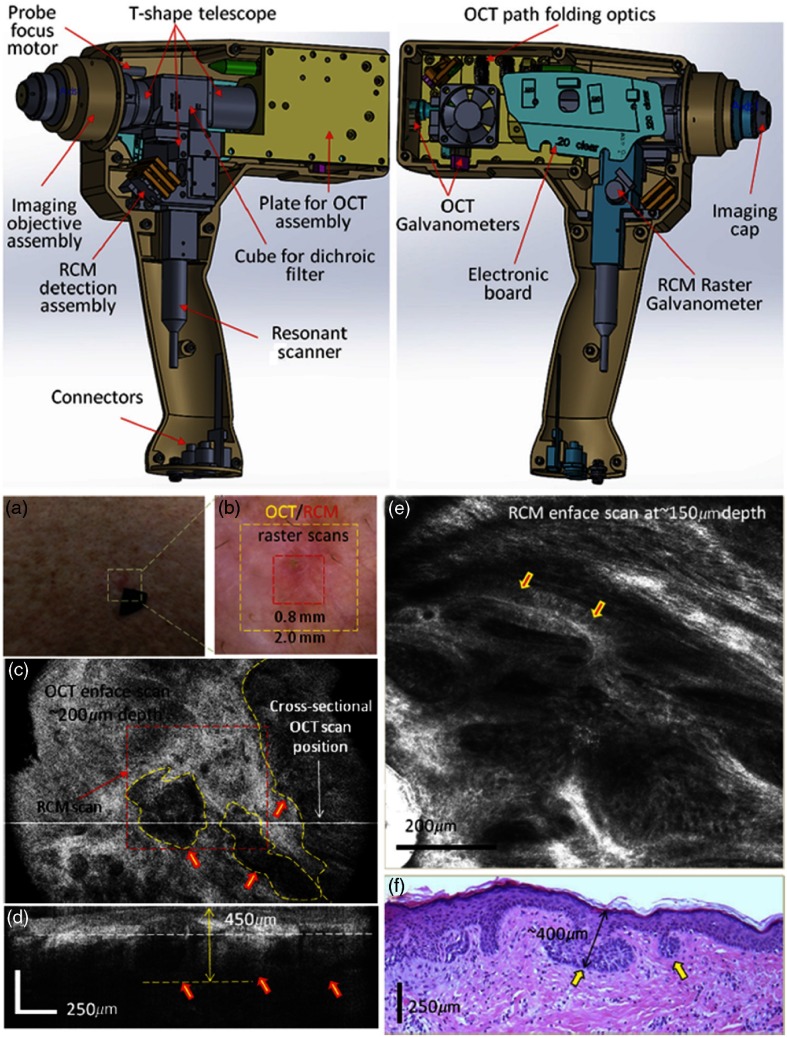
Handheld OCT-RCM probe. (Top) CAD design and schematic. (Bottom) (a) Clinical image of an erythematous macule. (b) Dermoscopy image of shiny white lines and serpentine vessels in the imaging region. (c) *En face* and (d) cross-sectional OCT images of hypoechoic areas (arrows), suggestive of BCC. (e) RCM showing cord-like structures with peripheral palisading (arrows) admixed with a fibrotic stroma, suggestive of BCC. (f) Histology of the lesion confirms superficial BCC, with multiple small tumor nests originating from the epidermis (H&E, 4×). Modified and reprinted with permission.^183^

Reflectance confocal microscopy (RCM) has also been combined with OCT in a handheld probe, as shown in Fig. 26.^183^ With this probe, it was possible to obtain volumetric OCT images of BCC, with enhanced cellular-level details at the selected depth of interest using RCM. A custom-made water immersion objective lens with an NA of 0.9 for RCM, and an NA of 0.1 when utilized for OCT, enabled a common path for the handheld probe. Detailed CAD drawings complement the imaging performance of this system, which is able to generate coregistered images of both cross-sectional tissue microstructures as well as *en face* cellular morphology of BCC *in vivo*.

**Fig. 27:**
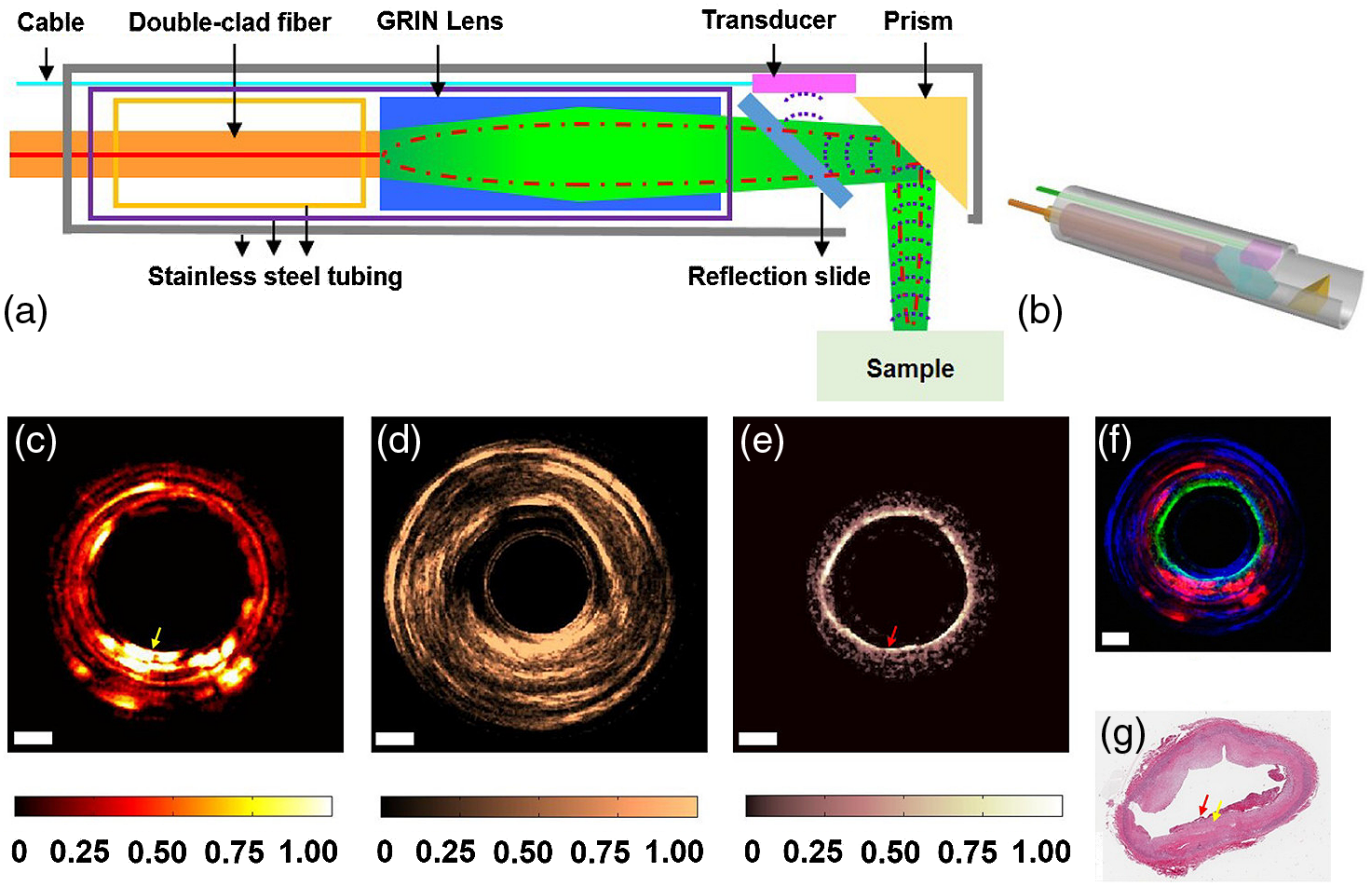
Integrated multimodal (OCT, US, and PAI) probe. (a) Schematic of distal end of probe and optical path. (b) CAD rendering of the probe. (Bottom) Cross-sectional images of human artery with atherosclerotic plaque. (c) PAI image, (d) US image, (e) OCT image, (f) color-coded RGB image, and (g) H & E histology. Reprinted (adapted) with permission from Ref. 186. Copyright (2016) American Chemical Society.

OCT provides greater imaging depth than most other optical imaging modalities, such as confocal microscopy or nonlinear optical imaging. Yet, when compared to nonoptical imaging modalities, such as US imaging, MRI, or x-ray CT, the reduced imaging depth is a limitation in many clinical applications. To circumvent this problem, OCT has been integrated with US for intravascular imaging.^184^^,^^185^ OCT was able to provide enhanced resolution of the vascular microstructures, while US was able to image deeper structures outside of the vessels. Notably, a single miniaturized endoscope capable of OCT, US, and photoacoustic imaging (PAI) has recently been developed for human skin and intravascular imaging,^186^ illustrated in Fig. 27. A dual-clad fiber was used to deliver light for PAI and OCT, and an integrated ultrasound transducer was used for US and PAI. The inherently coregistered images provide contrast from optical absorption, scattering, and the acoustic properties of tissue, although the imaging speed (15 s per Tri-B-scan) could be improved for more practical clinical *in vivo* imaging. The coregistered information from multiple modalities with multiple contrast mechanisms and varying penetration depths allows for greater investigation and characterization of disease states in endoscopic applications than a single modality.

## 6 Stage 3 Systems: OCT on the Go

**Fig. 28:**
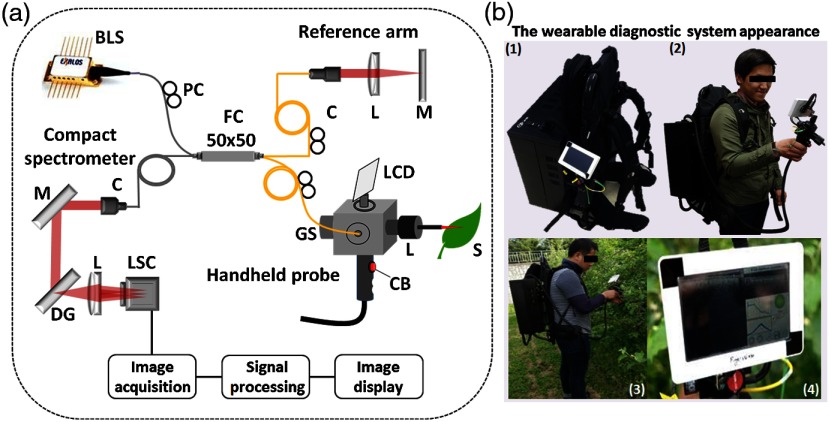
Backpack OCT system for crop QC. (a) Schematic of the portable, compact, and battery-powered backpack-OCT system. (b) (1) wearable system, (2) user wearing pack, (3) user scanning plant leaves, and (4) handheld LCD screen. Reprinted with permission.^187^

Not all OCT applications occur with access to stable power from a wall outlet. Furthermore, having a portable cart-based system does not guarantee that transit of the system to and from the imaging deployment site will be convenient or feasible for a single person to manage. Similar restrictions exist in factory or commercial quality control (QC) applications, where size and cost are also serious considerations. Food production QC is one area where grains/seeds, meats, and fruits/vegetables are often constantly monitored to identify and track production issues. Recently, the first portable battery-powered OCT system was demonstrated to characterize crop health in the field (Fig. 28).^187^ The system was designed to be completely self-contained in an 8-kg backpack form that could be easily carried. The battery platform powers everything needed for the OCT system, with a runtime of ∼6  h.

The system is unique in that its backpack unit houses all of the optical imaging and detection components, power supply, and computer control. The tethered handheld unit has a small LCD screen and can quickly visualize data for interpretation. The system has also been optimized to save power when not in use. Data are analyzed using a pass/fail classification algorithm that, with further testing in larger studies, could eventually allow the user to identify issues in real-time at the point of measurement, or similarly be used as an automated QC system for food or process manufacturing to identify issues and/or errors.

Engineers designing systems for use in QC^188^^,^^189^ or low-resource settings^190^^–^^193^ have to meet certain requirements for reliable and nearly continuous operation in daily practice. However, in various clinical scenarios^194^^–^^196^ or military applications in the field,^197^ users require an even more portable system that can be carried and utilized over a significant period of time in a wider variety of situations and use cases. Systems such as these can be brought to patients, rather than having to bring patients to instruments, which overcomes a significant hurdle that often exists in the delivery of more advanced healthcare.

## 7 Stage 4: Future Point-of-Care Devices

Handheld OCT systems have demonstrated comprehensive clinical utility for the early diagnosis of infection and disease. Unfortunately, no fully developed Stage 4 OCT systems (pocket-sized, self-contained) exist to date. Researchers are actively working to reduce the size and cost of OCT systems by considering alternate detection methods^198^^,^^199^ and light sources,^200^^,^^201^ as well as exploring costs and tradeoffs among various OCT platforms. For specific clinical imaging applications, it may be more practical to reduce the performance requirements of the OCT system to save costs and reduce system complexity and size, while still providing the needed data for clinical decision making.

The original configuration for OCT systems was named time-domain OCT (TD-OCT), given its detection scheme. Researchers have continued system development since inception, pushing the limits of these TD-OCT systems.^202^^,^^203^ However, TD-OCT systems have been used less frequently in the last decade because of the improved imaging speed and SNR afforded by Fourier-domain OCT (FD-OCT) systems.^35^^,^^204^ One important advantage of TD-OCT systems, however, is their potential cost advantage over FD-OCT systems, as they do not require expensive spectrometers or fast swept laser sources.

**Fig. 29:**
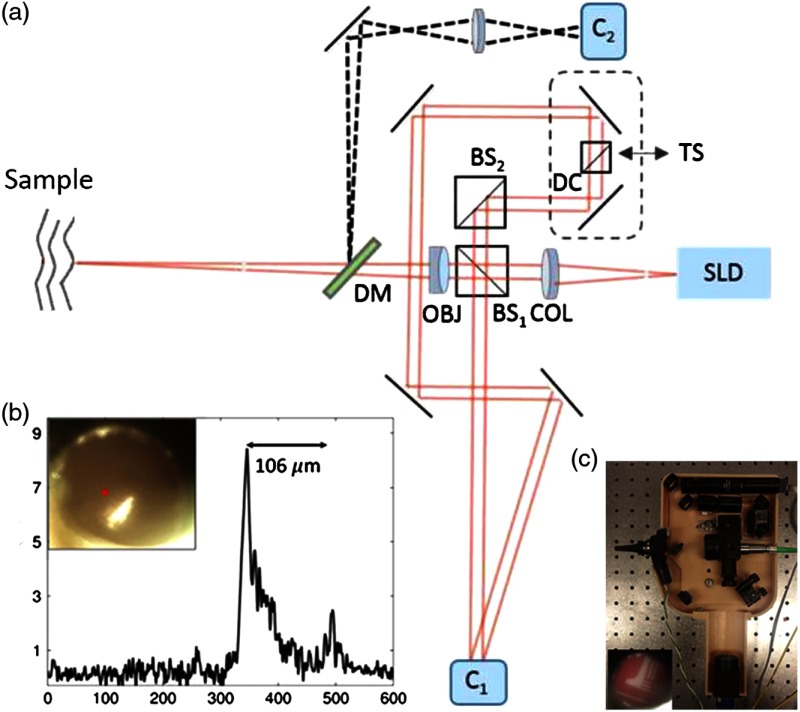
Reduced cost linear-OCT handheld probe for ear imaging. (a) Optical layout of handheld probe. (b) An averaged A-line from a TM recorded from an *in vivo* human subject. (c) Completed (cover removed) handheld LCI imaging probe. Abbreviations: SLD, superluminescent diode; COL, collimation optics; ${\mathrm{BS}}_{1}/{\mathrm{BS}}_{2}$, 50:50 beam splitter; OBJ, objective lens; DM, dichroic mirror; DC, dispersion compensation; TS, translation stage; $C_{1}/C_{2}$, 2-D CCD camera for recording interference and surface images. Reprinted with permission.^205^

Recently, an integrated handheld LCI system has been developed and demonstrated for *in vivo* ear imaging,^205^ based on the principle of linear-OCT.^206^ The overall cost to develop this system was significantly lower than portable cart-based FD-OCT systems, roughly by a factor of four.^81^ Other than the light source, all components needed for this initial system design were housed in a 3-D-printed handheld probe, as shown in Fig. 29. The measured axial resolution and sensitivity of the system were around 5.2  μm and 80 dB, respectively.

Multiple reference OCT (MROCT) systems are another low-cost TD-OCT technology, based on the optical system within a CD/DVD computer drive. This platform has the potential to enable a wide range of point-of-care and personal care applications.^207^ In MROCT, a depth scan is achieved by utilizing a partial mirror and actuator to generate a recirculating optical delay line, which extends the axial scan range. The overall cost and size of a typical MROCT system is anticipated to be similar to a CD/DVD unit, although these units are still in the early stages of development.^208^

Given the advantages of Fourier-based systems over TD-OCT, miniaturizing an FD-OCT system has great potential to be useful in clinical applications, especially in low-resource settings. Instead of utilizing standard spectrometers and CCD cameras, systems developed on a miniaturized silicon-based photonics platform can ideally utilize the cost-savings from mass production capabilities in the semiconductor industry. Recently, a commercial entity created an OCT system that retails for less than $10,000 USD.^209^ Although limited information is currently available on its design, this achievement was possible given the use of components manufactured at scale for other industries, paired with cost-reduction in other key OCT components such as a reflective parabolic mirror to collimate the light, a liquid lens to scan the beam over a sample, a “loop design” spectrometer, and a low-cost line-scan array detector used for scanning and reading bar-codes.

One research group created an integrated silicon-based arrayed-waveguide spectrometer,^210^ although it still required an external line-scan camera. Later, a “TriPleX” interferometer platform for SS-OCT was fabricated on a chip platform, which integrates a Michelson interferometer, sample and reference arms, and ports for external balanced photodiode detectors.^211^ Soon after, these two systems were combined to provide a combined high-quality silicon-based FD-OCT interferometer and spectrometer unit,^212^ which provided high-quality images of *in vivo* human skin with an SNR of 74 dB. Another system for SS-OCT integrated the complete dual-balanced quadrature detection system on a chip,^213^ with a detection sensitivity of 94 dB.

Often, these systems are unexpectedly limited by their compact size, as useful working distances for biomedical applications frequently require an external reference arm to properly match optical delay. To overcome this issue, one system added an on-chip long-path reference arm for SD-OCT systems,^214^ and later incorporated grating couplers to be compatible with SS-OCT systems.^215^ In both designs, the light source and spectrometer or detectors were separate from the integrated optical paths and interferometer. There are now efforts to fully integrate all of these components into a single integrated platform. Recently, an integrated platform for SS-OCT combined a significant amount of detector and interferometer components onto a single platform,^216^ although an external laser system was still required. Systems such as these aim to have a design that encourages a silicon-based fabrication process that can be scaled to commercial levels and significantly reduce the unit cost.

With the continual size reduction and improvement of these silicon-based OCT chips, as well as the expanding OCT ecosystem, it is feasible that an entire OCT system could one day be miniaturized into a fully contained battery-powered handheld package. Situations that will benefit enormously from this technical development of miniaturized OCT systems are users and subjects in remote areas where a decentralized, tiered, and distributed healthcare system exists, or in emergency situations where a quick observational tool could rapidly determine care in a time-critical environment. Similarly, cost-effective access to OCT imaging technology can only benefit future adopters of this technology, for any application.

## 8 Conclusion

In conclusion, only a subset of the many advances in handheld OCT technology that have evolved since the inception of OCT in the early 1990s have been discussed here. The new level of refinement of commercial systems, and even research systems, is a testament to the hard work and dedication of OCT researchers and engineers, as well as the many clinical partners who have driven the biomedical imaging applications of OCT. The availability of optical and hardware components from the commercial OCT ecosystem has also enabled advances out of laboratories that were once limited to design engineering firms. As OCT handheld probes continue to develop and mature, we expect the technology will find an even broader user base and serve as an indispensable tool for high-resolution, noninvasive, and nondestructive imaging.
